# Differential cell-type dependent brain state modulations of sensory representations in the non-lemniscal mouse inferior colliculus

**DOI:** 10.1038/s42003-019-0602-4

**Published:** 2019-09-30

**Authors:** Chenggang Chen, Sen Song

**Affiliations:** Tsinghua Laboratory of Brain and Intelligence and Department of Biomedical Engineering, Beijing Innovation Center for Future Chip, Center for Brain-Inspired Computing Research, McGovern Institute for Brain Research, Tsinghua University, Beijing, 100084 China

**Keywords:** Sensory processing, Midbrain, Multiphoton microscopy

## Abstract

Sensory responses of the neocortex are strongly influenced by brain state changes. However, it remains unclear whether and how the sensory responses of the midbrain are affected. Here we addressed this issue by using *in vivo* two-photon calcium imaging to monitor the spontaneous and sound-evoked activities in the mouse inferior colliculus (IC). We developed a method enabling us to image the first layer of non-lemniscal IC (IC shell L1) in awake behaving mice. Compared with the awake state, spectral tuning selectivity of excitatory neurons was decreased during isoflurane anesthesia. Calcium imaging in behaving animals revealed that activities of inhibitory neurons were highly correlated with locomotion. Compared with stationary periods, spectral tuning selectivity of excitatory neurons was increased during locomotion. Taken together, our studies reveal that neuronal activities in the IC shell L1 are brain state dependent, whereas the brain state modulates the excitatory and inhibitory neurons differentially.

## Abstract (web summary)

Chenggang Chen and Sen Song report a new method for imaging the first layer of the non-lemniscal inferior colliculus in awake mice. They show that neuronal activity in the inferior colliculus shell L1 is brain-state dependent.

## Introduction

Neuronal activity in the auditory cortex (ACx) is profoundly influenced by the brain state^1^. For example, sound evokes transient activity in the ACx at sound onset or offset under anesthesia^2^, whereas sustained activity is more prevalent under wakefulness^3,4^. Diverse receptive field changes were observed during sleep compared with wakefulness^5–7^. Furthermore, compared with the synchronized state, spectral and temporal tunings in the ACx were improved during the desynchronized state^8,9^. In contrast to findings in the ACx, neuronal activity in the auditory midbrain or inferior colliculus (IC) is generally thought to be brain state independent. For example, the spectral and temporal tunings of IC neurons in the awake animals are very similar to those in the anesthetized animals^10,11^. However, the conclusion may not hold true when different subregions of IC are considered.

IC consist of several subregions that participate in different pathways^12^. Sensory information is relayed by the lemniscal or driver pathway and the nonlemniscal or modulator pathway in parallel^13^. Recently, the nonlemniscal pathway has gained widespread attention. Neurons in the nonlemniscal pathway are not only more broadly tuned and display stronger stimulus adaptation but also project to the non-sensory brain areas for innate behaviors^14,15^. In the auditory system, the lemniscal pathway includes the IC core (central IC (ICC)), ventral subregion of medial geniculate body (MGBv), and primary ACx, the nonlemniscal pathway includes the IC shell (dorsal and external IC (ICD and ICX, respectively)), dorsal and medial subregion of MGB (MGBd and MGBm, respectively), and non-primary ACx^14^. In almost all the previous studies that compared tuning differences in the IC during anesthetic versus awake state, only the lemniscal IC core was investigated^16–18^, but see Duque and Malmierca^19^. Whether and how the brain state changes affect the auditory processing in the nonlemniscal IC shell is less well understood.

Neuronal activity in the cortex is also modulated by locomotion: tuning selectivity typically remains unchanged, response gains are affected, but the sign of the effect depends on modality. In ACx, the response gains are decreased^20,21^. In visual cortex (VCx), the gains are increased^22^. The mechanism of locomotion-related effects is still unclear. In the ACx, Zhou et al. found that the excitatory and parvalbumin-positive inhibitory neurons are inhibited by the cortical layer 1 (L1) neurons, while Schneider et al.^23^ reported that inhibitory neurons are excited by motor cortex, which in turn inhibits the excitatory neurons. In the VCx, Fu et al.^24^ reported that locomotion excites vasoactive intestinal peptide (VIP)-positive inhibitory neurons through cholinergic inputs, which inhibits somatostatin (SOM)-positive inhibitory neurons and disinhibit excitatory neurons, while Pakan et al.^25^ found that all inhibitory and excitatory neurons are excited, challenging the disinhibition model. IC is also composed of excitatory and inhibitory neurons, which receive distinct extrinsic inputs^12^. Therefore, it would be interesting to examine how the locomotion modulates the gains and selectivity of excitatory and inhibitory neurons and probe the candidate circuit mechanism in the subcortical IC.

To shed light on how brain states modulate the spontaneous and sound-evoked activities in the IC, we used in vivo two-photon calcium imaging to study the IC shell L1 in awake, behaving mice. We found that wakefulness increased the spectral and temporal tuning selectivity of excitatory neurons compared to those measured under anesthesia. Similarly, locomotion increased the spectral tuning selectivity of excitatory neurons compared to those measured under stationary periods overall. These results revealed that sound representations of the IC shell L1 excitatory neurons were enhanced during wakefulness and locomotion, highlighting the importance to study sensory processing in awake, behaving animals in subcortical sensory areas^26,27^.

## Results

### Calcium imaging in the IC shell L1 of behaving mice

**Fig. 1:**
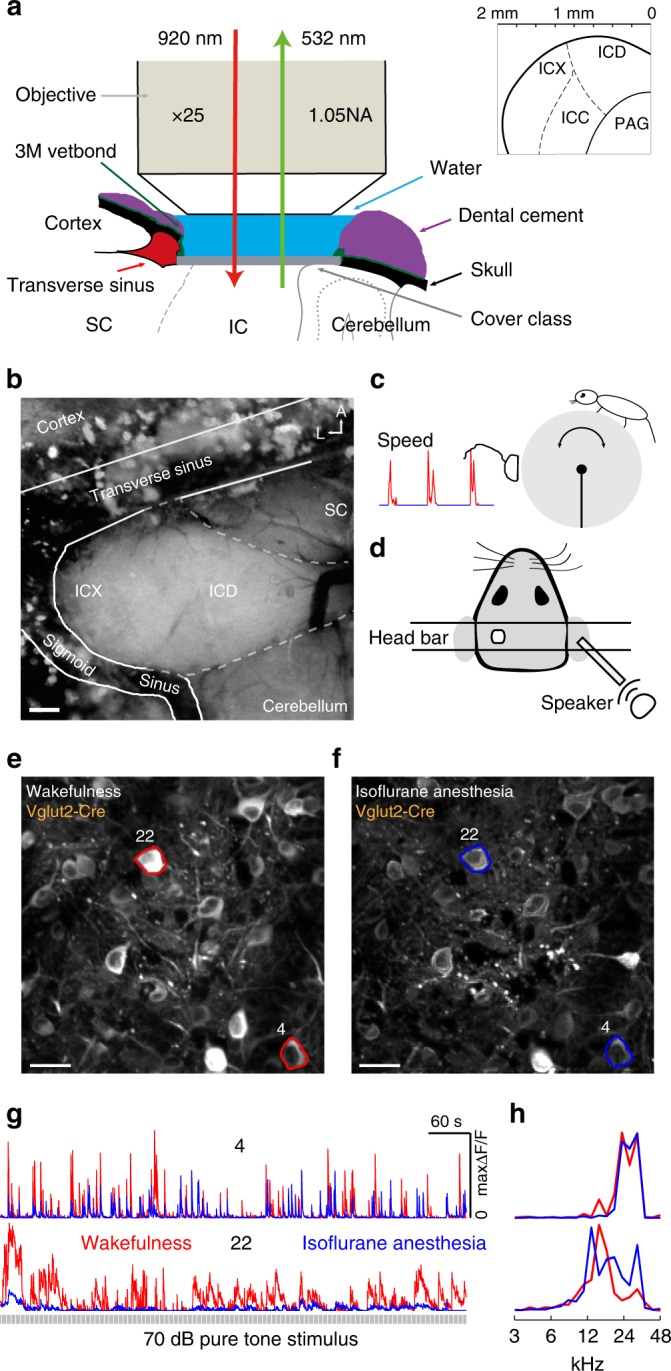
Chronic two-photon calcium imaging of neuronal activity in the IC shell L1 of awake, behaving mice. **a** Sagittal view showing the cranial window and the in vivo imaging diagram. For the detailed experimental procedures, see the text. Inset: Coronal view showing the central IC (ICC), ICD, ICX, and periaqueductal gray (PAG). **b** Horizontal view showing the anatomical location of dorsal IC (ICD) and lateral IC (ICX) and the neighboring superior colliculus (SC) and cerebellum. Notice that the IC could be identified by the deep white color. Scale bar: 150 μm. A anterior, L lateral. **c** Schematic of the experimental set-up. A mouse is head-fixed but free to run on a treadmill with the optical mouse to register its running speed. **d** Close-field pure tones and click train sound stimuli used. **e** Example field-of-view (FOV) in the IC shell L1 of awake Vglut2-Cre mouse. **f** Identical FOV in the IC shell L1 of 1% isoflurane-anesthetized Vglut2-Cre mouse. Stacked images along time axis (3000 images, 5 × 1 × 20 × 30, fps × ITI × stimuli × repeats) were used to generate the standard deviation (SD) figure. More active neurons will have larger SD and brighter color in the figure. 30 μm depth below the dura. Scale bar: 25 μm. **g** Example fluorescent traces of two excitatory neurons during wakefulness (red) and anesthesia (blue) when stimulated with 70 dB pure tones. **h** Corresponding spectral tuning curves during wakefulness and isoflurane anesthesia

To prevent the tissue regrowth and brain movement during awake recordings, we developed to our knowledge a novel method that enabled us to image the IC shell L1 in the awake, behaving mice (Fig. 1a). First, the shape of craniotomy and cover glass were not circular (Fig. 1b) but depended on the exposed area of IC, superior colliculus (SC), and cerebellum; second, the cover glass was put on the surface of IC, SC, cerebellum, and sigmoid sinus directly. No part of the skull bone and transverse sinus should be covered by the cover glass. During the awake recordings, the mouse was free to run on the treadmill. The locomotion and stationary periods were recorded using an optical mouse or rotation decoder (Fig. 1c). The mouse head was fixed using two parallel head bars. Neurons from the left IC were imaged, and sound stimuli were delivered to the right ear with the close-field speaker (Fig. 1d). IC is caudal to the SC, rostral to the cerebellum, and could be clearly identified by its deep white color. ICD was medial to the ICX, but no reliable histological proof could be used to differentiate ICD/ICX L1. Based on our in vitro histology (Supplementary Fig. 1) and in vivo imaging (Supplementary Fig. 2) evidence, we were probably recording responses from the dorsal part of IC shell L1, i.e., ICD L1 (see “Methods”).

With the above-described method, we could compare the same population of neurons during wakefulness (Fig. 1e) and isoflurane anesthesia (Fig. 1f). We compared the dynamic fluorescence traces (Fig. 1g) and frequency tuning curves (Fig. 1h) of two example neurons (regions of interest (ROIs) in Fig. 1e, f) during different states (red and blue in Fig. 1g, h). Neuronal activities (Δ*F*/*F*) during wakefulness were larger than those during isoflurane anesthesia (Fig. 1g). Was the brain state change-induced quantitative differences in the fluorescence accompanied by qualitative changes in auditory tuning? To address this question, we normalized the frequency tuning curves by their respective maximum values. Compared to isoflurane anesthesia, tuning selectivity of the number 4 neuron was similar, whereas tuning selectivity of the number 22 neuron was increased during wakefulness.

Taken together, our newly developed in vivo calcium imaging method enabled us to compare the auditory tunings in the IC shell L1 under different brain states.

### Isoflurane anesthesia broadens the bandwidth (BW) of excitatory neurons

The excitatory and inhibitory neurons in the IC receive different extrinsic inputs^12^. Therefore, the brain state changes might modulate the activity of excitatory and inhibitory neurons differently.

**Fig. 2:**
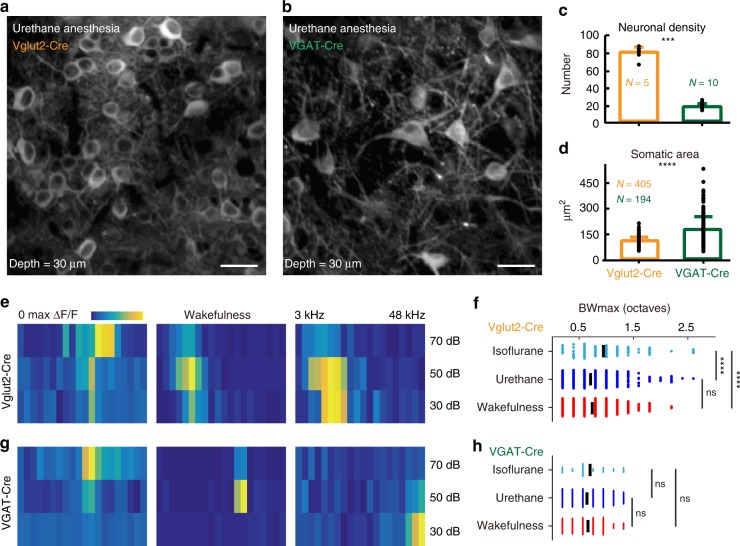
Isoflurane anesthesia decreases the spectral tuning selectivity of excitatory neurons. **a** Example FOV of excitatory neurons. **b** Example FOV of inhibitory neurons. Images are generated by projecting the image stacks along the *Z* axis with maximum intensity projection type. 30 μm depth below the dura. Scale bar: 25 μm. **c** The neuronal density (number of neurons per 160 × 160 μm^2^) of virus-infected neurons is higher in Vglut2-Cre than in VGAT-Cre mice. **d** The somatic area is larger in VGAT-Cre than in Vglut2-Cre mouse (*n* = 405 and 194 neurons from 15 FOVs of 15 mice for Vglut2-Cre and VGAT-Cre mice, respectively). Unpaired *t* test. Example frequency response area (FRA) of three excitatory neurons (**e**) and inhibitory neurons (**g**) when stimulated with pure tones (3–48 kHz, 0.2 octave per stimulus, 30, 50, and 70 dB). Maximum bandwidths (BWmax) of excitatory neurons (**f**) and inhibitory neurons (**h**) during isoflurane anesthesia (*n* = 79 and 28 neurons from 5 FOVs of 4 Vglut2-Cre and 5 FOVs of 4 VGAT-Cre mice, respectively), urethane anesthesia (*n* = 667 and 219 neurons from 36 FOVs of 13 Vglut2-Cre and 30 FOVs of 8 VGAT-Cre mice, respectively), and wakefulness (*n* = 510 and 113 neurons from 12 FOVs of 6 Vglut2-Cre and 8 FOVs of 6 VGAT-Cre mice, respectively). Kruskal–Wallis test with Dunn’s multiple comparisons test. ***p < 0.001, ****p < 0.0001, ns non-significant

To target glutamatergic excitatory neurons, we used the Vglut2-Cre line (Fig. 2a), as IC excitatory neurons express the Vglut2 but not Vglut1^28^. To target GABAergic inhibitory neurons, we used the VGAT-Cre line (Fig. 2b), as glycinergic neurons, which also express VGAT, are absent from the IC^29^. IC shell L1 comprises a unique sheet of neurons <100 μm below the pial surface^30^ (Fig. 2a, b). For example, at the 30 μm depth, the morphology of the excitatory neurons (Fig. 2a) and inhibitory neurons (Fig. 2b) could be clearly identified. GCaMP6f proteins were specifically expressed in the cytoplasm of both cell types. The density of GCaMP6f-expressed inhibitory neurons was consistent with the proportion of inhibitory neurons in the IC shell^31^, i.e., about three times more of excitatory neurons were labeled than the inhibitory neurons (*p* = 0.0007; 81 ± 6.3 versus 19 ± 2, number of neurons per 160 × 160 μm^2^; Mann–Whitney *U* test; Fig. 2c). Furthermore, the somatic areas of inhibitory neurons were highly heterogeneous and larger than the excitatory neurons (*p* < 0.0001; 230 ± 99.7 versus 142 ± 27.8; Mann–Whitney *U* test; Fig. 2d), which was similar to the previous studies^31,32^. Thus, in our studies, GCaMP6f seems to have labeled the inhibitory neurons in an unbiased fashion.

To compare the spectral tuning selectivity of excitatory and inhibitory neurons during wakefulness and anesthesia, we used pure tone stimuli with different frequencies and intensities to construct the frequency response area (FRA; Fig. 2e, g). The BW at 10–40 dB above threshold (i.e., BW10/20) is a generally used index for the width of FRA^33^. However, in this study, we only used three sound levels (30, 50, and 70 dB). Therefore, we compared the maximum BW of the FRA at any sound level (i.e., BWmax) for both types of neurons under different states. The BWmax has been used by previous ICD calcium imaging studies^30^. Higher spectral tuning selectivity means narrower BW. For the third excitatory neuron, the BWmax is 1.2 octave that was evoked by 50 dB sound (Fig. 2e). The distribution of sound intensity that evoked the BWmax was similar for both types of neurons (30 dB, Ex: 31.3%, In: 35.9%; 50 dB, Ex: 27.1%, In: 31%; 70 dB, Ex: 41.6%, In: 33.1%). The BWmax of excitatory neurons during isoflurane anesthesia, wakefulness, and urethane anesthesia is shown in Fig. 2f. The BWmax of excitatory neurons under isoflurane anesthesia were broader than those under the urethane anesthesia and wakefulness (*p* < 0.0001, isoflurane: 0.9494 ± 0.0072 octaves, urethane: 0.7442 ± 0.0006 octaves, wakefulness: 0.7659 ± 0.0007 octaves, Kruskal–Wallis test with Dunn’s multiple comparisons test) but were not different between the urethane anesthesia and wakefulness (*p* = 0.1381). Isoflurane mainly potentiates the inhibitory γ-aminobutyric acid type A (GABA_A_) receptors^34^, whereas urethane potentiates both the inhibitory GABA_A_ and excitatory *N*-methyl-d-aspartic acid receptor^35^. In the VCx, isoflurane reduces the direction selectivity by decreasing the mutual information of population neurons^36^. Different changes in the local network produced by isoflurane and urethane may explain their diverse effect over the spectral tunings of IC shell L1 excitatory neurons. Regarding the inhibitory neurons, the BWmax was similar under different brain states (*p* > 0.7988, isoflurane: 0.7143 ± 0.0130 octaves, urethane: 0.6667 ± 0.0016 octaves, wakefulness: 0.6832 ± 0.0028 octaves, Kruskal–Wallis test with Dunn’s multiple comparisons test; Fig. 2h).

One recent study that combined optogenetic and electrophysiology methods found that the frequency selectivity of IC excitatory and inhibitory neurons are similar in the anesthetized mouse^33^. It is challenging to record the neurons in the IC shell L1 with an electrode. Thus we believed that it is important to compare the spectral tuning characters between the excitatory and inhibitory neurons in the IC shell L1 during both anesthesia and wakefulness. The sound level that evoked the largest responses among all the stimuli (i.e., best intensity) is similar for both cell groups (30 dB, Ex: 28.5%, In: 27.7%; 50 dB, Ex: 31.9%, In: 28.3%; 70 dB, Ex: 39.6%, In: 44.0%). In addition to the best intensities, the best frequencies of both cell groups were similar in both the ultrasonic range (>20 kHz, Ex: 31.6%, In: 35.8%) and low-frequency range (<5 kHz, Ex: 29.5%, In: 27.6%).

Although the best intensities and best frequencies of excitatory and inhibitory neurons were similar, the BWs of their receptive fields were different. During the urethane anesthesia, compared to the excitatory neurons, the BWmax of inhibitory neurons were significantly narrower (*p* = 0.0009; Mann–Whitney *U* test). A similar difference was also observed when the BW20 was compared (*p* = 0.0043; 0.7010 ± 0.0016 octaves versus 0.6544 ± 0.0009 octaves; Mann–Whitney *U* test). During wakefulness, the inhibitory neurons were still more selective for the sound frequency (*p* = 0.0036; Mann–Whitney *U* test).

Altogether, in contrast to wakefulness, isoflurane anesthesia strongly broadened the BWmax of excitatory neurons (Fig. 2f). Furthermore, spectral tuning selectivity of the IC shell L1 excitatory neurons was significantly lower than the inhibitory neurons during both anesthesia and wakefulness.

### Urethane anesthesia decreases temporal tuning selectivity

**Fig. 3:**
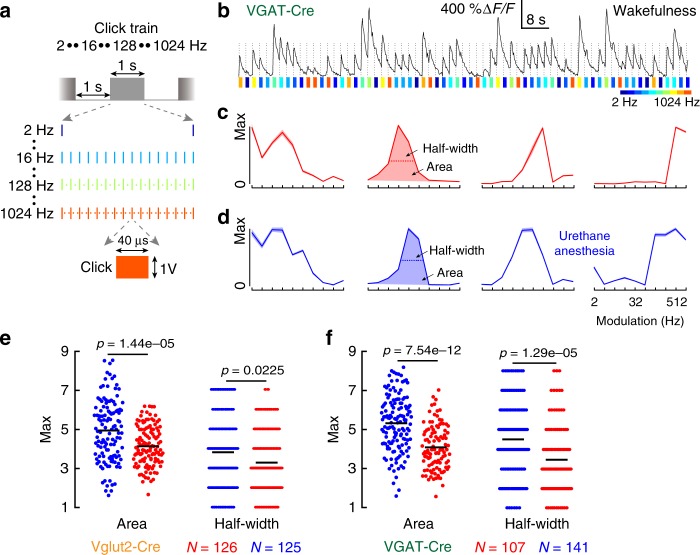
Urethane anesthesia decreases the temporal tuning selectivity of excitatory and inhibitory neurons. **a** Click trains (temporal modulation frequency: 2–1024 Hz) are used to characterize the temporal tuning. Repetition rate is 30 Hz. Inter-stimulus interval (ISI) is 2 s. **b** Snippets of a fluorescent change (black trace) of one inhibitory neuron during wakefulness when stimulated with click trains (color bars). The neuronal response was phase locked to the onset of sound stimuli (vertical dotted lines), but their magnitude depends on the specific modulation frequency. **c** Example temporal tuning curves of four inhibitory neurons during wakefulness. Responses are sorted with increasing temporal modulation frequency. All tuning curves are normalized to the same maximum values. **d** Example temporal tuning curves of four inhibitory neurons during urethane anesthesia. The half-width corresponds to the number of modulation frequency at half-peak amplitude. The area corresponds to the summarized responses below the tuning curve. Only the half-width and area of band-pass tuning curves are included for the following analysis. The low-pass (first one) and high-pass (last one) tuning curves are demonstrated but are not analyzed. **e** Tuning area and half-width of excitatory neurons during urethane anesthesia (blue) and wakefulness (red). **f** Tuning area and half-width of inhibitory neurons. Urethane anesthesia: 6 FOVs of 4 Vglut2-Cre mice and 5 FOVs of 4 VGAT-Cre mice. Wakefulness: 14 FOVs of 6 Vglut2-Cre mice and 13 FOVs of 6 VGAT-Cre mice

To compare the temporal tuning selectivity of excitatory and inhibitory neurons under wakefulness versus anesthesia, we used the click train stimuli with ten different modulation frequencies (Fig. 3a). The neuronal activities (i.e., fluorescent traces) were closely dependent on the different sound stimuli (Fig. 3b). For example, the first inhibitory neuron displayed low-pass tuning curve with the best modulation frequency (BMF) at 2 Hz, whereas the last inhibitory neurons displayed high-pass tuning curve with the BMF at 512 Hz (Fig. 3c, Supplementary Fig. 3). Temporal tuning selectivity was characterized only for the band-pass tuning curves using the half-width of tuning curves and the normalized area underlying the tuning curves (Fig. 3d), as the half-width and area were difficult to define for low- and high-pass tuning curves. Higher temporal tuning selectivity means narrower half-width and smaller area.

Second, we compared the temporal tuning half-width and area of excitatory neurons during wakefulness versus urethane anesthesia (Fig. 3e). The temporal tuning selectivity of excitatory neurons under wakefulness was significantly higher than those under urethane anesthesia (area, urethane: 4.9458 ± 0.0126, awake: 4.1378 ± 0.0080; half-width, urethane: 3.8080 ± 0.0145, awake: 3.2778 ± 0.0122; Mann–Whitney *U* test). Interestingly, similar trends were also observed for the inhibitory neurons (area, urethane: 5.3493 ± 0. 0.0379, awake: 4.1141 ± 0.0384; half-width, urethane: 4.5035 ± 0.0319, awake: 3.4673 ± 0.0324; Mann–Whitney *U* test; Fig. 3f).

Lastly, we compared the temporal tuning characteristics of the excitatory and inhibitory neurons in the IC shell L1. Under the urethane anesthesia, tuning selectivity of excitatory neurons were significantly higher than the inhibitory neurons (area, *p* = 0.0314; half-width, *p* = 0.0035; Mann–Whitney *U* test). Under wakefulness, the tuning selectivity of excitatory and inhibitory neurons were similar (area, *p* = 0.6958; half-width, *p* = 0.5695; Mann–Whitney *U* test).

In summary, urethane anesthesia decreased the temporal tuning selectivity of both excitatory and inhibitory neurons (Fig. 3e, f). Furthermore, the temporal tuning selectivity of the IC shell L1 excitatory neurons was significantly higher than the inhibitory neurons under urethane anesthesia.

### Locomotion modulates the spontaneous activities of IC neurons

**Fig. 4:**
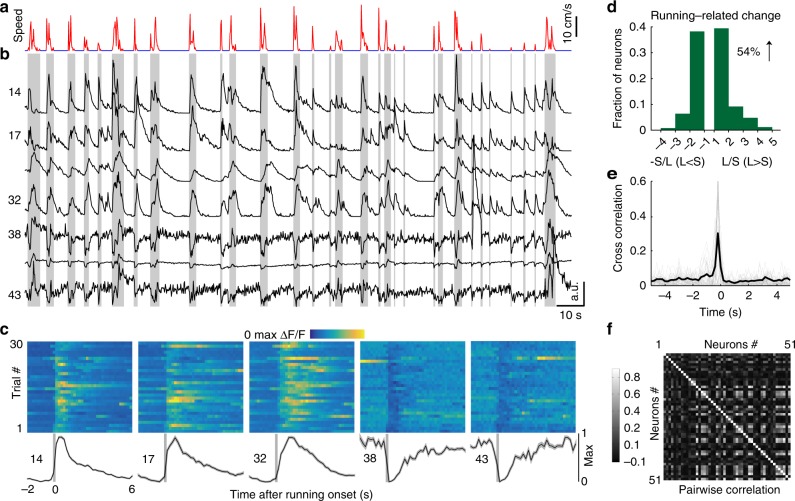
Locomotion enhances or suppresses the spontaneous activities of inhibitory neurons. **a** Locomotion (red) and stationary (blue) periods. **b** Example fluorescent traces of seven inhibitory neurons during locomotion and stationary periods. Gray shading indicates bouts of locomotion. Spontaneous activities of the first four neurons are enhanced by locomotion, whereas the last three neurons are suppressed by locomotion. **c** Spontaneous activities (2 s prior to locomotion and 6 s after locomotion) are plotted for the 30 individual running trials (color map) and averaged (black lines). Vertical gray bars show onsets of locomotion. **d** Across the populations, the spontaneous activities of 54% and 46% of inhibitory neurons are significantly enhanced and suppressed by locomotion, respectively (*n* = 102 neurons from 4 FOVs of 4 mice). L locomotion, S stationary. **e** Individual inhibitory neurons are highly correlated with locomotion. The thin gray lines are the cross-correlation curves of all recorded inhibitory neurons of the same FOV. The thick black curve is the average of all gray curves. **f** Strong pairwise correlations among the inhibitory neurons are observed during locomotion

The activities of cortical excitatory and inhibitory neurons are well known to be modulated by locomotion^25^. It is unknown whether locomotion could also modulate the activities in the midbrain. To address this question, we recorded the treadmill movement (Fig. 4a), along with the baseline fluorescent traces of multiple inhibitory neurons (Fig. 4b) simultaneously. In the Fig. 4b, activities of 7 example inhibitory neurons from the simultaneously imaged 51 neurons were demonstrated. The first four neurons were only excited during locomotion, whereas the last three neurons were mainly inhibited during locomotion. For example, the number 38 neuron primarily decreased its activities during the periods of locomotion but obviously increased its activities near the end, suggesting that it was predominately inhibited by other inhibited neurons. In Fig. 4c, the changes of activities across individual running onsets were demonstrated, which clearly showed the enhanced and the suppressed neuronal activities after running onsets. In all, 81% of inhibitory neurons were significantly modulated by locomotion. Across the locomotion-modulated neurons, 54% and 46% of neurons were enhanced and suppressed, respectively (Fig. 4d).

**Fig. 5:**
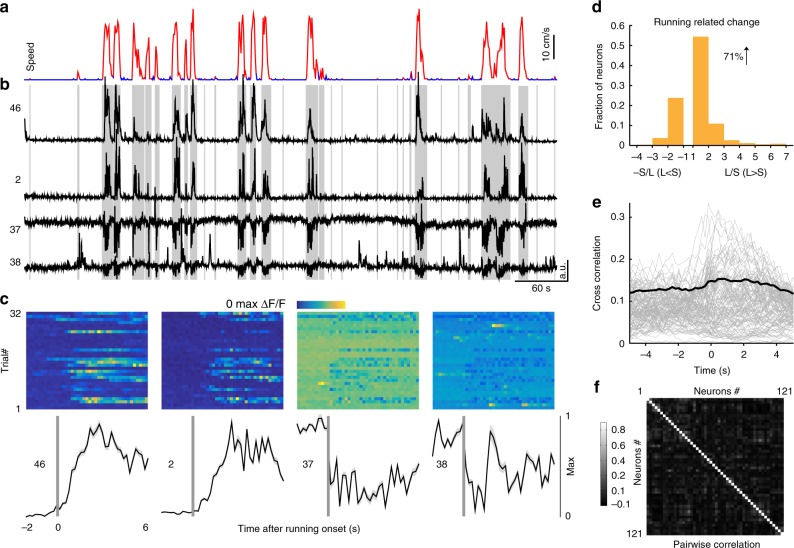
Spontaneous activities of excitatory neurons are less correlated with locomotion. **a** Locomotion (red) and stationary (blue) periods. **b** Example fluorescent traces of four excitatory neurons during locomotion and stationary periods. Gray shading indicates bouts of locomotion. Spontaneous activities of the first two neurons are enhanced by locomotion, whereas the last two neurons are suppressed by locomotion. **c** Spontaneous activities are plotted for the 32 individual running trails (color map) and averaged trails (black lines). Vertical gray bars show onsets of locomotion. **d** Across the populations, the spontaneous activities of 71% and 29% of excitatory neurons are significantly enhanced and suppressed by locomotion, respectively (*n* = 312 neurons from 6 FOVs of 6 mice). L locomotion, S stationary. **e** Individual excitatory neurons are weakly correlated with locomotion. **f** Weak pairwise correlations among the excitatory neurons are observed during locomotion

Similarly, we also recorded the treadmill movement and the fluorescent traces of multiple excitatory neurons simultaneously (Fig. 5a, b). In Fig. 5b, activities of 4 example excitatory neurons from simultaneously imaged 121 neurons were demonstrated. Compared to the stationary periods, during locomotion, the first 2 neurons were enhanced, whereas the last 2 neurons were suppressed (Fig. 5c). In all, 62% of excitatory neurons were significantly modulated by locomotion. Across the locomotion-modulated neurons, 71% and 29% of neurons were enhanced and suppressed, respectively (Fig. 5d).

Potential noise confounds during locomotion may affect the results. To rule out this possibility, we have performed additional analysis and control experiments (Supplementary Fig. 4). First, the noise was not correlated with locomotion (raw noise versus shuffled noise, *p* = 0.90), and the correlation values were smaller than the correlation values of neuronal activities of inhibitory and excitatory neurons with locomotion (*p* < 0.001, Kruskal–Wallis test; Supplementary Fig. 4a). Second, the sound levels during two states were quite similar for the frequency >3 kHz (*p* = 0.89; locomotion: 18.9 ± 0.08 dB, stationary: 18.5 ± 0.05 dB, Mann–Whitney *U* test; Supplementary Fig. 4b). Third, locomotion-related neuronal activities still preserved and correlated with locomotion (*p* = 0.47, Mann–Whitney *U* test) when masked by a 75-dB white noise sound (3–80 kHz flat response; Supplementary Fig. 4c). Thus our results were unlikely to be an artifact of the self-generated sound noise.

Altogether, our results demonstrated that locomotion could enhance or suppress the activities of IC shell L1 inhibitory and excitatory neurons.

### Activities of inhibitory neurons are more correlated with locomotion

Although the activities of inhibitory and excitatory neurons were both modulated by locomotion (Figs. 4c and 5c), the temporal dynamics of this modulation effect was different. For example, neuronal activities of the first four inhibitory neurons were nearly synchronized with the onsets of locomotion (Fig. 4b). In contrast, neuronal activities of the first two excitatory neurons lagged the onsets of locomotion (Fig. 5b, c).

To characterize the temporal dynamics of neural activities with locomotion and among the simultaneously recorded neurons, we calculated the cross-correlation and pairwise-correlation values, respectively. The cross-correlation values between the fluorescent traces of inhibitory neurons and running speed revealed a single positive peak at −200 ms, signifying that the running onset was ahead of the spontaneous neuronal activities (Fig. 4e, 0.31 ± 0.03). In contrast, cross-correlation values between the calcium signals of excitatory neurons and running speed do not have a significant peak (Fig. 5e, 0.1457 ± 0.0005). Although the cross-correlation values are small, the excitatory neurons are still correlated with and modulated by locomotion, as the shuffling results revealed small and smooth cross-correlation values and no neurons were modulated by locomotion (Supplementary Fig. 5a). As a population, the pairwise correlation values of both excitatory (Fig. 4f, 0.0975 ± 0.0002) and inhibitory (Fig. 5f, 0.0331 ± 0.00005) neurons were low, but more highly correlated pairs were observed in the inhibitory neurons (In > 0.3: 12.4%, Ex > 0.3: 0.33%).

We compared the cross-correlation and pairwise-correlation values among the locomotion-enhanced and locomotion-suppressed inhibitory neurons (In-En, In-Su), and locomotion-enhanced and locomotion-suppressed excitatory neurons (Ex-En, Ex-Su). The In-En neurons were highly correlated with locomotion, when compared with the In-Su, Ex-En, and Ex-Su neurons (In-En 0.39 ± 0.14, *N* = 86; In-Su 0.17 ± 0.10, *N* = 74; Ex-En 0.14 ± 0.11, *N* = 193; Ex-Su 0.15 ± 0.07, *N* = 80; *p* < 0.0001, *p* < 0.0001, *p* < 0.0001; Kruskal–Wallis test with Dunn’s multiple comparisons test, Supplementary Fig. 5b). Moreover, the In-En neurons were highly correlated with each other during locomotion, when compared with the In-Su, Ex-En, and Ex-Su neurons (In-En 0.18 ± 0.013, *N* = 1049; In-Su 0.06 ± 0.003, *N* = 498; Ex-En 0.03 ± 0.00001, *N* = 6142; Ex-Su 0.03 ± 0.0007, *N* = 3215; *p* = 0.0013, *p* < 0.0001, *p* < 0.0001; Kruskal–Wallis test with Dunn’s multiple comparisons test, Supplementary Fig. 5c).

Taken together, the activities of locomotion-enhanced inhibitory neurons were more correlated with locomotion (Supplementary Fig. 5b) and more correlated with each other (Supplementary Fig. 5c) than the locomotion-inhibited inhibitory neurons and excitatory neurons.

### Locomotion decreases the BW of excitatory neurons

**Fig. 6:**
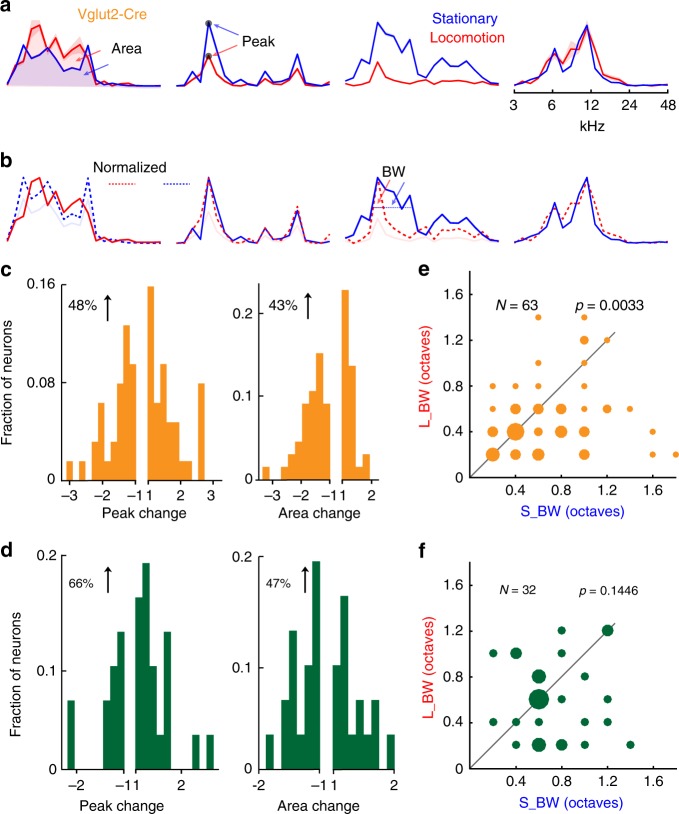
Locomotion increases the spectral tuning selectivity of excitatory neurons overall. **a** Spectral tuning curves of four example excitatory neurons during the stationary periods (blue) and locomotion (red). The peak corresponds to the maximum value of averaged responses. The area corresponds to the summarized responses below the tuning curve. **b** The tuning curves of peak-inhibited neurons in one state are normalized to the same peak amplitude of the other state. Red or blue dashed lines represent the normalized tuning curves. **c** The modulation effects of locomotion on the peak and area of excitatory neurons. **e** Spectral bandwidths of excitatory neurons during locomotion (L_BW) and stationary periods (S_BW). The dot diameter is proportional to the number of neurons (10 FOVs of 5 mice). **d**, **f** Same figures as in **c**, **e** but for the inhibitory neurons (10 FOVs of 6 mice). Wilcoxon signed-rank test for all comparisons

Previous studies revealed that locomotion increases the visually evoked responses in the layer 2/3 (L2/3) of VCx and visual thalamus^22,37,38^ but attenuates sound-evoked responses in the L2/3 of ACx and MGB^20,23,39^. However, it is still unknown whether the locomotion could also modulate the auditory responses in the midbrain. To address this question, we compared the spectral tuning curves during locomotion versus stationary periods (red versus blue; Fig. 6, Supplementary Figs. 6, 7, and 8; Supplementary Data 2 and 3). In Fig. 6a, the spectral tuning curves of 4 example excitatory neurons when stimulated with 70 dB pure tones were demonstrated. During locomotion, the maximum response of the first neuron was mildly enhanced, the third neuron was severely suppressed, and the fourth neuron remain unchanged. In summary, 48% excitatory neurons increased their peak amplitudes after running onsets (Fig. 6c). In addition to the peak amplitudes, we also compared the area (i.e., overall responses under stimuli) underlying the tuning curves. In summary, 43% excitatory neurons increased areas after running onsets (Fig. 6c). Compared to the excitatory neurons, 66% and 47% inhibitory neurons increased their peak amplitudes and areas after running onsets, respectively (Fig. 6d).

Previously, we found that the awake versus anesthetic state changes could modulate the auditory processing of excitatory neurons (Fig. 2f). Thus it would be interesting to examine the auditory processing during locomotion versus stationary periods. In Fig. 6b, the spectral tuning curves during locomotion (red) and stationary periods (blue) were normalized to the same peak amplitudes. Compared to the stationary periods, tuning selectivity of the third and fourth neurons was increased and decreased during locomotion, respectively. In summary, the spectral BWs of excitatory neurons during locomotion were significantly narrower than those during the stationary periods (Fig. 6e, stationary: 0.6794 ± 0.0061 octaves, locomotion: 0.5048 ± 0.0049 octaves, *p* = 0.0033, Wilcoxon signed-rank test). In contrast, spectral BWs of inhibitory neurons during the locomotion were like those during the stationary periods (Fig. 6f, stationary: 0.7188 ± 0.0094 octaves, locomotion: 0.6000 ± 0.0103 octaves, *p* = 0.1446, Wilcoxon signed-rank test). In general, 48% of excitatory and 44% of inhibitory neurons increased and 24% of excitatory and 28% of inhibitory neurons decreased their spectral tunings selectivity.

Overall, locomotion does not affect the response gain of the excitatory and inhibitory neurons to pure tone stimuli (Fig. 6c, d). Compared with inhibitory neurons, tuning selectivity of excitatory neurons was significantly increased (Fig. 6e, f).

### Locomotion does not affect temporal tuning selectivity

**Fig. 7:**
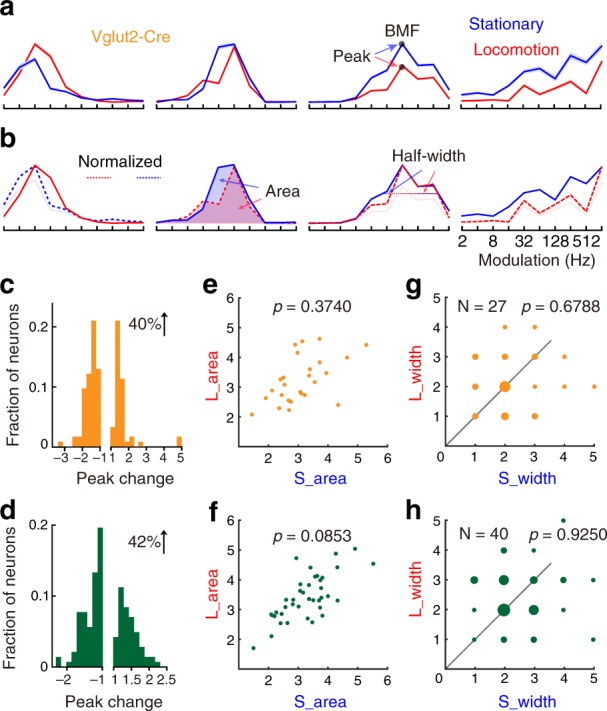
Locomotion does not affect the temporal tuning selectivity of excitatory and inhibitory neurons overall. **a** Temporal tuning curves of four example excitatory neurons during the stationary periods (blue) and locomotion (red). Only the band-pass tuning curves are analyzed for the tuning half-width and area. The peak corresponds to the maximum value of averaged responses. BMF corresponds to the modulation frequency that elicits the maximum responses. **b** The tuning curves of peak-inhibited neurons in one state are normalized to the same peak amplitude of the other state. Red or blue dashed lines represent the normalized tuning curves. The area corresponds to the summarized responses that were normalized below the tuning curve. The half-width corresponds to the number of modulation frequency at half-peak amplitude. **c** The modulation effects of locomotion on tuning peak amplitude of excitatory neurons. **e** Tuning area of excitatory neurons during locomotion (L_area) and stationary periods (S_area). **g** Tuning half-width during locomotion (L_width) and stationary periods (S_width). **d**, **f**, **h** Same figures as in **c**, **e**, **g** but for the inhibitory neurons. Twenty-two FOVs of 6 Vglut2-Cre mice and 13 FOVs of 6 VGAT-Cre mice (includes 5 types of rMTF)

Previously, we found that anesthetic state decreased the temporal tuning selectivity of both types of neurons (Fig. 3e, f). Therefore, we compared the temporal tuning curves during the locomotion versus stationary periods (red versus blue; Fig. 7, Supplementary Figs. 8, 9, and 10; Supplementary Data 4 and 5). In Fig. 7a, temporal tuning curves of four example excitatory neurons when stimulated with click train stimuli were demonstrated. During locomotion, the maximum response of the first neuron was enhanced, and the fourth neuron was suppressed. In summary, 40% excitatory neurons and 42% inhibitory neurons increased their peak amplitudes (Fig. 7c, d). Although the auditory responses were suppressed or enhanced during locomotion, the BMF was almost unchanged (Supplementary Fig. 11). Some neurons changed their types of temporal tuning curves (Supplementary Fig. 10c), but the overall proportion during locomotion and stationary periods was similar (Supplementary Table 1).

After normalizing their peak responses, temporal tuning selectivity during the locomotion versus stationary periods was compared (Fig. 7b). During locomotion, the temporal tuning selectivity of excitatory neurons were not significantly different than those during the stationary periods (area, stationary: 3.0687 ± 0.0313, locomotion: 3.2038 ± 0.0284, Fig. 7e; half-width, stationary: 2.2593 ± 0.0379, locomotion: 2.1481 ± 0.0336, Fig. 7g; *p* = 0.6788, Wilcoxon signed-rank test). Similarly, temporal tuning selectivity of inhibitory neurons during locomotion were like those during the stationary periods (area, stationary: 3.2570 ± 0.0206, locomotion: 3.4147 ± 0.0188, Fig. 7f; half-width, stationary: 2.5500 ± 0.0259, locomotion: 2.5250 ± 0.0271, Fig. 7h; *p* = 0.9250, Wilcoxon signed-rank test). In general, 33% of excitatory and 35% of inhibitory neurons increased and 30% of excitatory and 35% of inhibitory neurons decreased their temporal tuning selectivity.

Altogether, locomotion mainly decreases the temporal responses of excitatory and inhibitory neurons to click train stimuli (Fig. 7c, d). Overall, temporal tuning selectivity of the excitatory and inhibitory neurons was not affected by locomotion (Fig. 7e–h).

## Discussion

In contrast to other animal species, part of the SC and IC are exposed in the mouse. Therefore, in vivo calcium imaging or two-photon-guided patch-clamp recordings have been conducted in the mouse SC^40–42^ and IC^30,43,44^ in a handful of studies. However, only one prior study used the awake preparation in the mouse SC^45^. In that study, to minimize the movement artifact, a triangular silicone plug that bonded to a cover glass was positioned over the craniotomy. In our experience, we found that it was technically challenging to make the silicone plug without any bubbles inside. Furthermore, the transmittance of silicone was inferior to the glass and water. In contrast, in our method, only a single cover glass was positioned over the tissue directly (Fig. 1a), without extra preparation or sacrificing the transmittance. We believe that our method will be applicable to calcium imaging studies in both the IC shell and SC.

In the cortex, the L2/3 excitatory neurons respond more selectively to stimulus features than the inhibitory interneurons. In contrast, the L5 excitatory and inhibitory neurons both respond to a broad range of stimuli^46–48^. In this study, we found that the IC shell L1 inhibitory neurons have narrower spectral BW than the excitatory neurons during both anesthesia and wakefulness (Fig. 2f, h). What might be the source of the high-frequency selectivity seen in the IC shell L1 inhibitory neurons? Using two-photon-guided whole-cell recording, Geis and Borst revealed that the IC shell L1 inhibitory neurons that have large somas receive shorter first-spike latency excitatory inputs than other neurons^49^. Using cell-type-dependent monosynaptic rabies virus tracing, Chen et al. found that inhibitory neurons of the IC shell receive a higher proportion of ascending ventral cochlear nucleus inputs than the excitatory neurons^12^. Multipolar cells in the ventral cochlear nucleus project to the IC and are sharply tuned for sound frequency^50^. Therefore, we propose that the high-frequency selectivity of IC shell L1 inhibitory neurons is inherited from the ascending brainstem.

It is worth noting that inhibitory neurons in the mouse SC superficial layers are also more direction selective than the excitatory neurons^41^, and the origin of the direction selectivity is the retina^51^. Inhibitory neurons in the IC shell and SC superficial layers project to the nonlemniscal thalamus and non-sensory brain areas^12,52,53^. Therefore, inhibitory neurons in the colliculus may play a key role in inhibiting the first-order target and disinhibiting the second-order target with high feature selectivity.

Compared to wakefulness, we found that urethane anesthesia did not affect the spectral tuning selectivity (Fig. 2f, h), but the temporal tuning selectivity (Fig. 3e, f) of both excitatory and inhibitory neurons were decreased. To understand the effect of anesthesia over the auditory processing in the IC, three factors need be considered.

First, different subregions of IC exhibit different responses. Unlike IC shell, sound-evoked responses in the IC core are similar during anesthesia and wakefulness^10,11,17^. Second, different anesthetics have different effects. Our results showed that urethane anesthesia did not affect the spectral tuning selectivity, but the isoflurane anesthesia had the effect. Third, different stimuli evoke different responses. In the IC shell L1, the spectral tuning selectivity was not affected during urethane anesthesia, but the temporal tuning selectivity was significantly decreased.

For future studies performed in the IC shell that use the temporal-related sound stimuli, such as the amplitude-modulated signals, dynamic moving ripple, and natural sounds, the anesthetized preparation should be used with caution. Notice that popularly used Rayleigh statistics or vector strength in the temporal processing were not applicable to calcium imaging data due to its limited temporal resolution^47,54^. Therefore, the differences in temporal tuning we have observed are suggestive but should not be taken as conclusive evidence. In the future, two-photon-guided electrode recording could be applied to decipher the tuning properties of IC shell L1 cell-type-specific neurons under different brain states with higher temporal resolution^49^.

In this study, we found that the activities of locomotion-enhanced inhibitory neurons were highly correlated with locomotion (Fig. 4c), whereas other inhibitory neurons were suppressed. Similarly, the activities of VIP-positive inhibitory neurons in the cortex are also strongly correlated with locomotion, whereas SOM-positive inhibitory neurons are suppressed during locomotion^24,55,56^, but see Pakan et al.^25^. Thus subtypes of IC shell L1 inhibitory neurons may have been enhanced or suppressed by locomotion like those observed in the cortical inhibitory neurons.

Unlike the visual thalamus, sound-evoked responses in both the MGBv and MGBd are inhibited during locomotion^37^. Circuit mechanism of the effects of locomotion in the MGB is still unclear. Here we propose that the locomotion-enhanced IC shell inhibitory neurons may contribute to this effect in the MGBd. One distinctive feature of the IC is that 40% of the thalamic projection neurons are inhibitory neurons and the inhibitory inputs always leads the excitatory inputs^52^. Furthermore, as inhibitory projection neurons are characterized with a large somatic area and axosomatic excitatory terminals, they are considered to be one critical subtype of IC inhibitory neurons^31,57^. Therefore, during locomotion, neuronal activities in the MGBd are likely to be inhibited and gated by the ascending tectothalamic inhibitory projecting neurons. Further investigation is needed to identify the subtype of IC shell inhibitory neurons that faithfully convey locomotion information to the MGB.

For the amplitude or gain of tuning curves, about half of the excitatory and inhibitory neurons increased their responses when stimulated with pure tones and click trains under locomotion (Table 1). In contrast, nearly all the excitatory neurons of the ACx^20,23,39^ and the MGB^37^ were inhibited by locomotion.

**Table 1:** Summarized results of cell-type-specific, brain state-dependent neuron activities in the IC shell L1

	Excitatory neurons	Inhibitory neurons
Neuronal density	High	Low
Somatic area	Small	Large
Spectral tuning selectivity	Isoflurane < Urethane Isoflurane < Wakefulness Urethane ~ Wakefulness	Isoflurane ~ Urethane Isoflurane ~ Wakefulness Urethane ~ Wakefulness
Temporal tuning selectivity	Urethane < Wakefulness	Urethane < Wakefulness
Loco. enhancement	71%	54%
Cross-correlation with loco.	Low	High
Pairwise correlation during loco.	Low	High
Spectral tuning enhancement	Peak amplitude: 48% Summed activities: 43%	Peak amplitude: 66% Summed activities: 47%
Spectral tuning selectivity	Overall: increase Increase: 48% Decrease: 24% Preserved: 28%	Overall: unchanged Increase: 44% Decrease: 28% Preserved: 28%
Temporal tuning enhancement	Peak amplitude: 40%	Peak amplitude: 42%
Temporal tuning selectivity	Overall: unchanged Increase: 33% Decrease: 30% Preserved: 37%	Overall: unchanged Increase: 35% Decrease: 35% Preserved: 30%

**Fig. 8:**
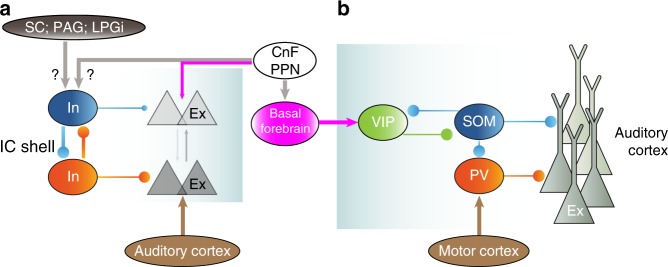
Neural circuit diagram based on current functional results, our previous anatomical results in the IC shell, and others’ results in the auditory cortex. **a** In the IC shell, one group of inhibitory neurons exhibit much higher cross-correlation values with locomotion than the other group of inhibitory neurons and excitatory neurons, which imply that they receive directly excitatory inputs from locomotion-related nuclei, mesencephalic locomotor region (MLR). MLR includes cuneiform nucleus (CnF) and pedunculopontine nucleus (PPN). Excitatory neurons also receive MLR inputs, but their in vivo activities are likely to be excited or inhibited by locomotion indirectly. The SC, PAG, and lateral paragigantocellular nucleus (LPGi) are also related with locomotion control. Compared with the excitatory neurons, the inhibitory neurons are highly inter-connected. Anatomically, the auditory cortex mainly innervates excitatory neurons. **b** In the auditory cortex, the inhibitory PV neurons receive long-range excitatory inputs from motor cortex, which in turn inhibit the local excitatory neurons. Locomotion-related MLR excites the basal forebrain, which in turn excite the inhibitory VIP neurons of auditory cortex. The VIP neurons will inhibit the inhibitory SOM neurons, which in turn disinhibit the local excitatory neurons. The MLR-related disinhibitory pathway also exist in the visual cortex, but the motor cortex-related inhibitory pathway is unique to the auditory cortex. Importantly, the net effect of locomotion over auditory cortex is inhibitory, since almost excitatory neurons are inhibited during locomotion

For the selectivity of spectral processing, the effects of locomotion were diverse: half of excitatory and inhibitory neurons increased, but one quarter of neurons decreased. For the selectivity of temporal processing, similar percentage of excitatory and inhibitory neurons increased, decreased, or preserved. At the population level, locomotion only significantly increased the spectral tuning selectivity of excitatory neurons (Table 1). In contrast, the spectral tuning selectivity of ACx neurons was well preserved^20^. Distinct long-range and local circuits of IC shell and ACx may explain the different effects of locomotion (Fig. 8a, b; Supplementary Note).

Our findings not only complement recent studies which show that sensory processing in the cortex are dynamically modulated, and extend such results to IC, but also give the hope that cortical studies could benefit from research in the colliculus in a comparative fashion. Laminar structure and cell types of IC shell are much simplified than the cortex, making it easier to decipher the connectivity and coding strategy of different cell classes (Fig. 8). Findings in the IC shell, in turn, might help shed light on similar computations carried out in cortex by carefully analyzing their similarities and differences.

## Methods

### Virus injection surgery

All the experiments were approved and conducted under the conformity to the Tsinghua University Animal Care and Use Committee. For all the experiments, 25 Vglut2-Cre (JAX: 028863, Jackson Laboratory, Bar Harbor, ME, USA) and 24 VGAT-Cre (JAX: 028862) adult mice with C57BL/6J genetic background (postnatal day 2–3 months) of both sexes were used. The mice were group housed with a reversed light cycle (12 h–12 h) and all the recordings were performed during the dark period.

The mice were intraperitoneally anesthetized with 40 mg/kg pentobarbital (dissolved in 0.9% saline; Solarbio, Beijing, China), and eye ointment was used to prevent eye drying during the surgery. Three alternative washes of Betadine and 70% (vol/vol) alcohol were applied on the head skin to prevent inflammation. The skin over the IC was cut by sterile scissors and forceps, after which the animals were mounted on a stereotaxic holder, and a part of the occipital bone was thinned at a diameter of ~1 mm with a 0.5-mm drill bit. When the boundary of IC could be clearly identified from the transverse sinus, cerebellum, and SC, the drilling was stopped, and a 26-gauge needle was used to penetrate and break the thinned skull at the desired location for remaining virus injection. AAV2/1.Syn.Flex.GCaMP6f.WPRE.SV40 (UPenn Vector Core) was diluted 1:1–5 in saline. Barnstedt et al. revealed that the IC shell comprises a unique sheet of neurons <100 μm below the pial surface^30^. Furthermore, too much neuropils’ fluorescence caused by GCaMP6f-expressed neurons within the deep area could contaminate the effective signals. As a result, we injected the AAV virus carrying the calcium indicator GCaMP6f into the ~50 μm superficial layer of IC of Vglut2-Cre and VGAT-Cre mice. In order to inject the virus, we used the micro-syringe pump (Micro4, WPI, Sarasota, FL, USA) and corresponding glass pipette. In order to penetrate the dura without pushing pressure, the pipette was pulled (P-1000, Sutter, Novato, CA, USA), cut (MF-830, Narishige, Tokyo, Japan), and ground (EG-401, Narishige) to 30 μm inner diameter and 45° inclination angle. After the virus injection, cyanoacrylate glue (Vetbond, 3M, Saint Paul, MN, USA) was applied to the incised skins and 0.1 mg/kg buprenorphine (Melone Pharma, Dalian, China) was intraperitoneally injected.

### Cover glass implantation

Ten days after the virus injection surgery, the mouse was anesthetized and the eye ointment, Betadine, and 70% alcohol was applied as described above. A large area of the skin was cut off, and the exposed periosteum was air dried and removed by low-speed drilling. The occipital bone over the transverse sinus (TS), sigmoid sinus, ICD, part of the ICX, SC, and cerebellum (Fig. 1b) was thinned with a 0.5-mm drill bit. Since heartbeat and locomotion could induce large amplitude of movement over TS, which would indirectly affect the IC when glass cranial window was placed over both TS and IC, the bone over TS was thinned but still fully covered the TS. To remove the thinned bone over sigmoid sinus, IC, SC, and cerebellum, a 26-gauge needle was used to break the bone over the junction of IC and cerebellum. When the bone was broken, a tweezer was used to peel off the bone.

In the traditional method, a single cover glass or a cover glass with a glass plug was placed above the craniotomy^58,59^. However, owing to the irregular shape of craniotomy, uneven surface of the dorsal midbrain, and two large blood vessels, this method does not apply to the IC suitably. Inspired by one two-photon study in the cerebellum^60^, we placed the cover glass over the brain tissue, instead of the craniotomy. In order to place the tiny cover glass over IC shell, we used one hand to gently push down the cover glass with a 26-gauge needle, and the other hand to add an extremely small volume of cyanoacrylate glue by the tip of another 26-gauge needle. The tiny cover glass was selected from the debris of standard 24 × 40 mm cover glass (thickness: 0.15 mm, Warner Instruments, Hamden, CT, USA). We have successfully imaged the neuronal somas and axonal boutons in the IC shell L1 with this method up to 4 months. Lastly, all the exposed skull was covered by a thin layer of cyanoacrylate glue and dental cement (Nissin, Kyoto, Japan), along with two parallel and 1-cm spaced tungsten head bars (1 mm diameter and 5 cm length; https://gwjsyxgs.taobao.com/). When the dental cement was cured, we applied a thin layer of cyanoacrylate glue to the surrounding area of the cranial window. This procedure could prevent the water leaking out from the gap between two-photon objective and glass cranial window. After glass implantation surgery, all the mice were allowed to recover for >2 weeks before imaging, including isoflurane anesthesia, urethane anesthesia, and wakefulness.

### Histology verification of the imaging area

Previously, in vivo IC imaging-related studies have inconsistent criteria on the ICD. Geis et al. recorded at 0.8 mm lateral from midline^43^, Barnstedt et al. imaged the whole IC dorsal surface^30^, and Ito et al. imaged the medial half side of dorsal IC^44^. The deeper layers of ICD and ICX were different, but their L1 was both composed of small flat cells and fiber bundles, which were parallel to the brain surface. Our imaging depth was <100 μm, which was located within ICD/ICX L1. Since no reliable histological proof could be used to differentiate ICD/ICX L1, instead of using the term “ICD L1,” we used the term “IC shell L1.” Here we showed some evidence (see below) that our recorded areas were likely to be the dorsal part of IC shell L1, i.e., ICD L1.

Initially, ICD and its four layers were defined by their different neuronal morphology within IC^61–63^. Based on the morphology of our GCaMP6f-expressed neurons (Supplementary Fig. 1), we could also identify the candidate ICD subregion and delineate different layers. Surface area within 100 μm depth was mainly composed of small flat cells and their fiber bundles were parallel to the brain surface. This area was likely to be ICD L1. In the deeper area, the commissural bundles were obvious. This area was likely to be ICD Layer3. In the deepest area, the dendrites of multipolar neurons extended perpendicular to ICC laminae were outstanding. This feature was typical for ICD Layer4. Three layers were defined in the ICX. ICX L1 was a continuation of ICD L1. In contrast with the ICD Layer3 that has obvious commissural bundles, ICX L2 composed of small neurons that aggregated in dense clusters^64^. In our GCaMP6f-expressed neurons, we did not observe clusters. Thus the virus was probably expressed within the candidate ICD subregion. In Supplementary Fig. 2 and Supplementary Data 1 (same mouse as Supplementary Fig. 1), three candidate ICD L1 neurons were directly excited during locomotion.

In summary, our imaged areas were within IC shell L1. Based on our in vitro histology and in vivo imaging evidence, we were probably recording responses from the ICD L1.

### Acoustic stimuli

Acoustic stimuli were generated with a custom software (LabVIEW, National Instruments, version: 8.6, Austin, TX, USA), which controlled the data acquisition card (PCIe-6321, National Instruments, maximum analog outputs update rate: 900 kHz, resolution: 16 bits). The generated acoustic stimuli were connected to BNC adaptor (BNC-2110, National Instruments) and feed to speaker drivers (ED1, Tucker-Davis Technologies, Alachua, FL, USA). To deliver the contralateral sound stimuli, we placed a 10-cm straight silicone tube that coupled to an electrostatic speaker (EC1, Tucker-Davis Technologies) along the inter-aural axis at the entrance of mouse’s right ear. The speaker with the coupled tube was calibrated using a microphone (GRAS, Holte, Denmark, type: 40BE), amplifier (Brüel & Kjær, Nærum, Denmark, type: 2610), running software, and processor (SigCal and RZ6, Tucker-Davis Technologies). We have also used this microphone to measure background noise during mice locomotion. Owing to the internal thermal noise, this microphone only works for sound level >40 dB. We found the sound level under locomotion and stationary period were similar and could not be identified from baseline noise level. Since we only used 70 dB sound stimuli or 75 dB masking noise during locomotion experiment, the high-frequency noise with no more than 40 dB will unlikely affect our conclusions.

With regarding to the spectral tuning experiment, a sequence of pure tones (50 ms duration, 5 ms onset, and 5 ms offset ramps) with 20 logarithmically spaced frequencies, randomly spreading from 3 to 48 kHz (4 octaves) with intensities of 30/50/70 dB sound pressure level (SPL) were presented. For the experiment that compared the spectral tunings during the stationary periods versus locomotion, only the 70 dB SPL pure tones were used. Each stimulus was broadcasted per second and repeated 30 times. Since there are 20 frequencies, 30 repeats, and 1-s inter-stimulus interval, each trial will need 10 min.

With regard to the temporal tunings experiment, we have used the 15 linear spaced (i.e., 10, 20, 30, 40, 50, 60, 70, 80, 90, 100, 200, 300, 400, 500, 600 times per second) and 10 logarithmically spaced (i.e., 2, 4, 8, 16, 32, 64, 128, 256, 1024 times per second) temporal modulation frequencies. Each single stimulus is 40 μs click train of 1 V amplitude (equal to 75 dB SPL of 4.6 kHz pure tone). Thus 1024 clicks will be presented in the 1024 Hz temporal modulation frequency (Fig. 3a). Each temporal modulation frequency is 1-s duration, repeated 30 times. Inter-stimulus interval is 2 s. Since there are 15/10 frequencies, 30 repeats, and 2-s inter-stimulus interval, each trial will need 15 (for 600 Hz) or 10 (for 1024 Hz) minutes.

### Imaging

During awake recordings, the mouse was head-fixed and placed on a 15 × 10 cm (diameter × width) cylinder treadmill. The coupled silicone tube was placed at the entrance of external ear and glued to the head bar with cyanoacrylate glue in case of dropping off during locomotion. The locomotion was detected with a closely placed USB-optical mouse (Logitech, M100r) near the side of cylinder treadmill or a rotation decoder (Freescale, Austin, TX) in the central rod of treadmill. The treadmill connected with the central rod by two smooth bearings. During the anesthesia recordings with isoflurane, a custom-build respiration mask was fitted to the head-fixed behaving mouse after the awake recordings. Using this method, we could compare the same field of view (FOV) between wakefulness and anesthesia. Isoflurane 0.8–1.2% (vol/vol) (RWD, Shenzhen, China) in pure oxygen (O_2_) was used. The breathing rate was in the range of 100–120 breaths per minute. Airflow from the isoflurane anesthesia mask might be a candidate noise source. Thus we measured the sound levels during isoflurane and urethane anesthesia, and no difference could be observed. During the anesthesia recordings with urethane, 1.6 g/kg urethane (dissolved in 0.9% saline; Sigma-Aldrich, St. Louis, MO, USA) was intraperitoneally used for the initial anesthesia and regularly supplied in 30-min interval with 0.4 g/kg urethane to maintain a stable level of anesthesia throughout the recording. The body temperature was maintained at 37 °C (rectal) using a closed loop DC temperature controller (FHC, Bowdoin, ME, USA).

Images were acquired with a custom-built two-photon microscope that was controlled by the open source ScanImage^65^ (http://scanimage.org, version: 3.8). 920 nm excitation light for GCaMP6f imaging from mode-locked Ti:Sapphire laser (Mai Tai eHP, Spectra-Physics of Newport, Santa Clara, CA, USA) was scanned by paired galvanometers (6215 H, 3 mm silver-coated mirror, Cambridge Technology, Lexington, MA, USA) and guided through a water-immersion objective (XLPLN25XWMP, ×25, 1.05 NA, Olympus, Tokyo, Japan). The laser power was controlled with the combination of a half-wave plate and a Glan-Laser polarizer (WPH10M and GL10B, Thorlabs, Newton, NJ, USA). Since the imaging depth is <100 μm, the laser power was typically <30 mW. Emitted fluorescence was collected with a dichroic mirror (FF665-Di02, Semrock, Rochester, NY, USA), a band-pass filter (FF01-527/70, Semrock), and a photomultiplier tube (R9880, Hamamatsu, Japan). The above detection parts along with the preamplifiers were enclosed within the commercial multi-photon detection module (2PIMS-2000-40-20, Scientifica, Uckfield, UK). The ambient noise was kept low by keeping the laser’s power supply and cooling unit in a separate room and enclosing them within a sound-attenuation chamber (http://www.sjjyn.net/, Beijing, China). The image acquisition was triggered by the rising edge of the acoustic stimulus. Image was scanned at 5 Hz as a series of 256 × 200 pixel images. The largest FOV is 320 × 320 μm (i.e., zoom factor = 1). The smallest FOV used was 160 × 160 μm (i.e., zoom factor = 2). The FOV was adjusted (i.e., zoom factor between 1 and 2) to cover more neurons and less blank areas. The average imaging depth was 40 μm, with a maximum depth of 90 μm. For some mice, lots of neurons could be clearly observed after 5 μm of depth. About 1–4 FOVs near the medial part of IC shell L1 (Supplementary Fig. 2a) was recorded in each mouse (*Z* axis step depth >25 μm). The FOV number was determined by the virus expression and cranial window quality.

In our experiments, we used the urethane anesthesia first, then used isoflurane anesthesia, and at last, we transited our protocols from anesthesia to awake recording. Therefore, most of mice used in the anesthetized preparations were sacrificed after each day’s experiment. The results shown in Fig. 1e–h were from the identical population of neurons of the same animal (awake recording first). The results shown in Fig. 3c–d were from the same animal, but the FOV was uncertain (awake recording first). In summary, for the comparison between anesthesia and wakefulness, >90% percentage of neurons were from different animals.

For the locomotion and stationary period state-related imaging, each FOV includes 3 sessions (spectral, temporal—1024 Hz, and temporal—600 Hz) or 4 sessions (spontaneous, spectral, temporal—1024 Hz, and temporal—600 Hz). To minimize the photobleaching effect, we waited at least 10 min before next imaging sessions. For each session, four types of file will be generated for further analysis: sound stimuli, stack images, locomotion, and pupil video.

### Images analysis

For the calcium imaging data, the lateral (*x*–*y*) movement artifact induced by the locomotion was corrected with TurboReg, a plugin of ImageJ (version: 1.48; http://imagej.nih.gov/ij/). Image stacks were registered (mode: rigid body) to the frame without any movement artifact. Elliptical ROIs were determined manually in the Matlab (Mathworks, Natick, MA, USA) and the fluorescence signals of somas were extracted and neuropil signals were subtracted using a custom software^45^. ROIs were selected by visually inspecting the images stack and selecting neurons that showed at least one fluorescence transient. The out-of-focus neuropils’ signals could contaminate the desired somas’ signals, which could potentially bias the auditory tunings of the somas^45^. The true signal was *f* = *R* − (*r* × *n*), where *R* was the raw fluorescence signal, *n* was the contamination signal (10 μm ring around the somas), and *r* was the contamination factor. To determine the value of *r*, we identified the horizontal blood vessel (i.e., *f* *=* 0) and recorded the raw signal *R* and contamination signal *n*. Therefore, the *r* was equal to the ratio of *R* and *n*, which ranged between 0.5 and 0.7 in different FOVs. The *r* values in the Vglut2-Cre and VGAT-Cre mice were similar. Cells with filled nuclei were excluded from data analysis.

The baseline fluorescence *f*_0_ was estimated using the iteration procedure^66^. Briefly, the mean and standard deviation of the ROI trace were estimated, then any data points with >1.5 standard deviations from the ROI trace were removed, and we repeated the procedures until no further deviated points were found. The ROI traces were normalized with *f*_0_, namely, (*f* *−* *f*_0_)/*f*_0_. Lastly, the non-negative deconvolution method^67^ was used to estimate the spike events. The *Z* axis standard deviation-projected image was generated using ImageJ (Image-Stacks-Z project-Projection type: Standard Deviation), then the same minimum and maximum displayed values were used (Image-Adjust-Brightness/Contrast-Set) for the awake and isoflurane-anesthetized mice.

### Data analysis

Each sound stimulus in each trail is random, and the locomotion of mouse is also random. The sound stimulus will be classified as locomotion type if locomotion happens during the 50 ms (spectral) or 1000 ms (temporal) sound stimuli. In Supplementary Figs. 6b, 7b, 9b, and 10b, the coincidence of sound stimuli with locomotion was demonstrated. In Supplementary Fig. 8, statistics of mice behaviors that included neurons which were tuned under both stationary periods and locomotion was demonstrated.

With regard to the spectral tuning experiment, neurons demonstrating auditory tunings were included if they exhibited a statistically significant difference (*p* < 0.01, one-way analysis of variance (ANOVA)) in the responses among the 20 tone frequencies across 30, 50, and 70 dB for FRA or only 70 dB for the locomotion experiment^30^. The best frequency was determined by the sound frequency that evoked the largest averaged response across all the sound levels. The threshold was the smallest SPL that exhibited a statistically significant difference (*p* < 0.01, one-way ANOVA) across the 20 tone frequencies. In order to quantify the spectral tuning width, responses below the half-peak amplitude were assigned to zero, and the remaining area that composed of logarithmically discrete frequencies was defined as FRA^30^. BW20 was defined as the BW 20 dB above the threshold. BWmax was defined as the maximum FRA BW at any SPL (anesthesia versus wakefulness) and the BW at 70 dB SPL (stationary periods versus locomotion).

With regard to temporal tuning experiment, neurons were included for analysis only if they exhibited a statistically significant difference (*p* < 0.01, one-way ANOVA) in the responses among the 10/15 frequencies. BMF was defined as modulation frequency that evoked the largest averaged response across the frequencies. The rate modulation transfer function (rMTF) was constructed from the summarized neuronal activities within the 1-s sound stimulation. After normalizing the rMTF by its maximum firing rate, we evaluated the shape of rMTF exactly as the method used by Ono et al.^33^. From 0 to max, we set 3 boundaries: 25%, 50%, and 75%. From 2 to 1024 Hz modulation frequency, there will be 9 steps. Next, we will exam the ratio of value changes of each sequential step. If the ratio is <25%, this step will be defined as STAY. If the ratio is >25% and is positive, this step will be defined as UP. If the ratio is >25% and is negative, this step will be defined as DOWN. Therefore, each step will be assigned as STAY, UP, or DOWN. There are 5 types of rMTF: low-pass, high-pass, band-reject, band-pass, and multi-peak. If there is no UP in the 9 steps, this rMTF will be low-pass; if there is no DOWN, it will be high-pass. If there is only one transition from DOWN to UP, it will be band-reject; if there is only one transition from UP to DOWN, it will be band-pass. All others will be multi-peak. For the 15 linear spaced temporal modulation frequency, there will be 14 steps and the shape evaluation of rMTF will be identical.

To characterize the temporal tuning selectivity, we calculated the half-width and area of rMTF. Only the band-pass type of rMTF is included for the tuning width and area analysis. The temporal tuning half-width was defined as the number of discrete frequencies at the half-peak amplitude; tuning area was defined as the summation of averaged response across the frequencies that normalized by the largest averaged response. Similar methods were used to measure the intracellular subthreshold responses^54^ and sustained calcium responses^4^. For the 15 linear spaced temporal modulation frequency, we changed the frequency to logarithmical scale, then analyzed the tuning width. Since we have only 7 pairs of excitatory and 11 pairs of inhibitory neurons that are both tuned during locomotion and stationary period using the linear spaced temporal frequency (Supplementary Table 1), we only show the results of 10 logarithmically spaced temporal modulation frequency.

### Statistics and reproducibility

For the locomotion-enhanced and locomotion-suppressed spontaneous neuron activities (Figs. 4d and 5d), the deconvolution “oopsi” values from Δ*F*/*F* were grouped as “locomotion” (L) and “stationary period” (S). Parametric unpaired *t* test was used to examine whether the locomotion-related neuron activities were significantly different from activities under stationary periods (*p* < 0.01). For those neurons that were significantly modulated by locomotion, the ratio of L to S was demonstrated. If the activity of L was larger than S, the ratio equals to L divided by S (L > S, ratio = L/S, enhanced). If the activity of L was smaller than S, the negative reciprocal of ratio was used (L < S, ratio = −S/L, suppressed). For the locomotion-enhanced and locomotion-suppressed sound-evoked neuron activities (Figs. 6c, d and 7c, d), parametric unpaired *t* test was used to examine whether the locomotion- and stationary period-related neuron activities were significantly different during sound stimulation (*p* < 0.01). Then the ratio of those significantly tuned neurons were demonstrated.

For statistical analysis, parametric unpaired *t* test, nonparametric unpaired Mann–Whitney *U* test, and nonparametric paired Wilcoxon signed-rank test were used for two samples and nonparametric Kruskal–Wallis test was used for three samples. For the comparison between anesthesia and wakefulness, the sample size of each state was different, therefore unpaired Mann–Whitney *U* test was used; for the comparison between locomotion and stationary periods, the sample size of each state was identical, therefore paired Wilcoxon signed-rank test was used. Data were reported as mean ± standard error of mean (SEM).

### Reporting summary

Further information on research design is available in the Nature Research Reporting Summary linked to this article.

## Supplementary information

Supplementary Information

Description of Additional Supplementary Files

Supplementary Data 1

Supplementary Data 2

Supplementary Data 3

Supplementary Data 4

Supplementary Data 5

Reporting Summary

## Data Availability

Data that support the findings of this study are available from the first author or corresponding author upon reasonable request. Raw data used to generate the Supplementary Figures can be found in Supplementary Data 1–5 files accompanying this manuscript.

## Code availability

Custom codes that support the findings of this study are available from the first author upon reasonable request.
