# Supplementary material for: Differential cell-type dependent brain state modulations of sensory representations in the non-lemniscal mouse inferior colliculus
Source: Commun Biol. 2019 Sep 30;2:356. doi: 10.1038/s42003-019-0602-4 (PMC6769006; doi:10.1038/s42003-019-0602-4)
Supplement: Supplementary file 1 — Supplementary Information [file 42003_2019_602_MOESM1_ESM.pdf]

Supplementary Figures

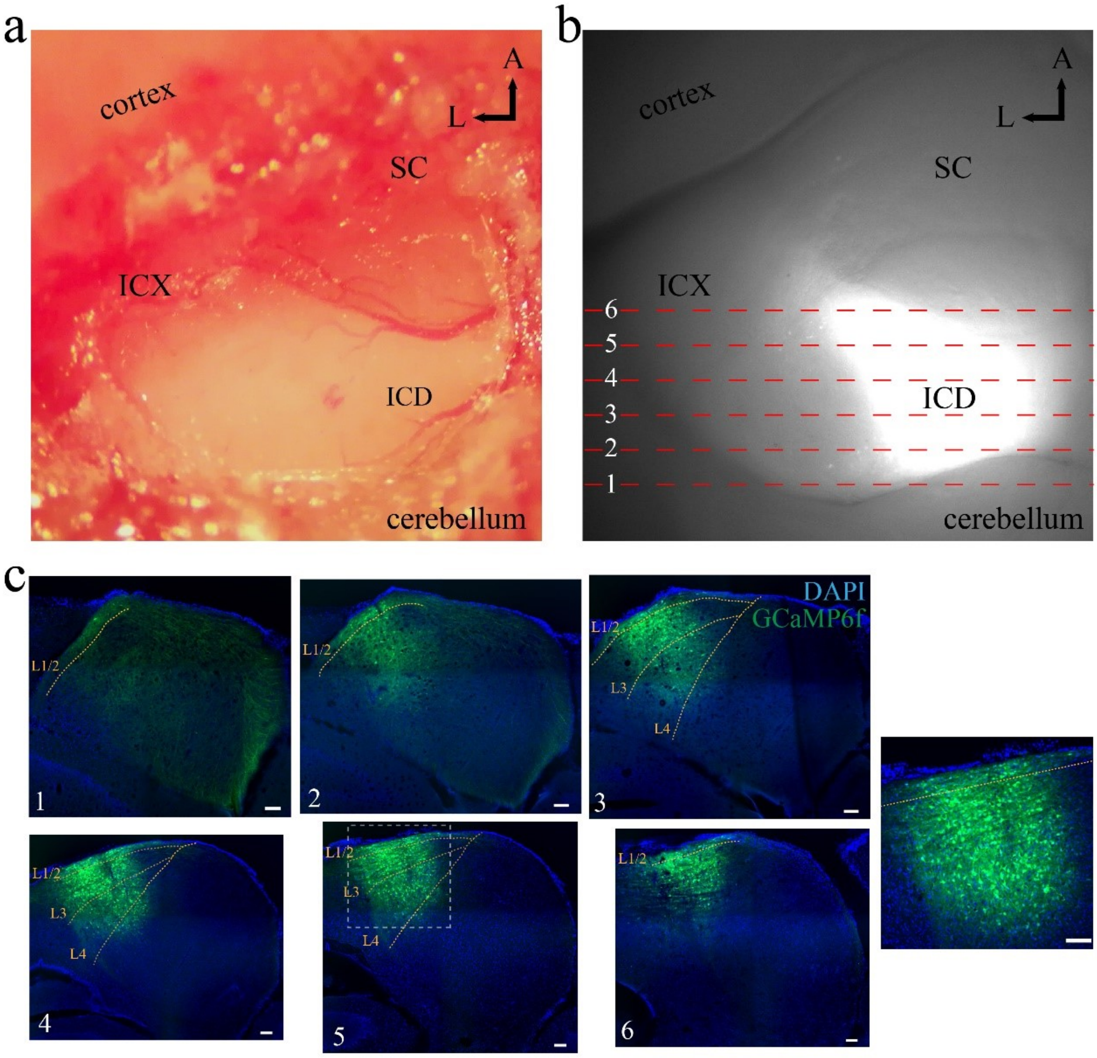

Supplementary Figure 1. The GCaMP6f virus was probably expressed in the candidate ICD L1. (a) FOV in the stereoscope after the glass window implanting surgery. (b) FOV in the upright fluorescence microscope using the PFA fixed brain. (c) The left side of IC was sliced along the posterior to anterior direction, stained using DAPI and imaged using confocal microscope. Noticed that the virus expression area was restricted within the candidate ICD subregion. Specifically, layer1/2 is mainly composed of small flat cells and their fiber bundles are parallel to the brain surface, layer3 has obvious commissural bundles and in the layer4, the dendrites of multipolar neurons extend perpendicular to ICC laminae are outstanding. The ICX is distinguished by its unique layer2 that composed of clusters. In our GCaMP6f expressed neurons, we did not observe clusters. Scale bar: 100 μm.

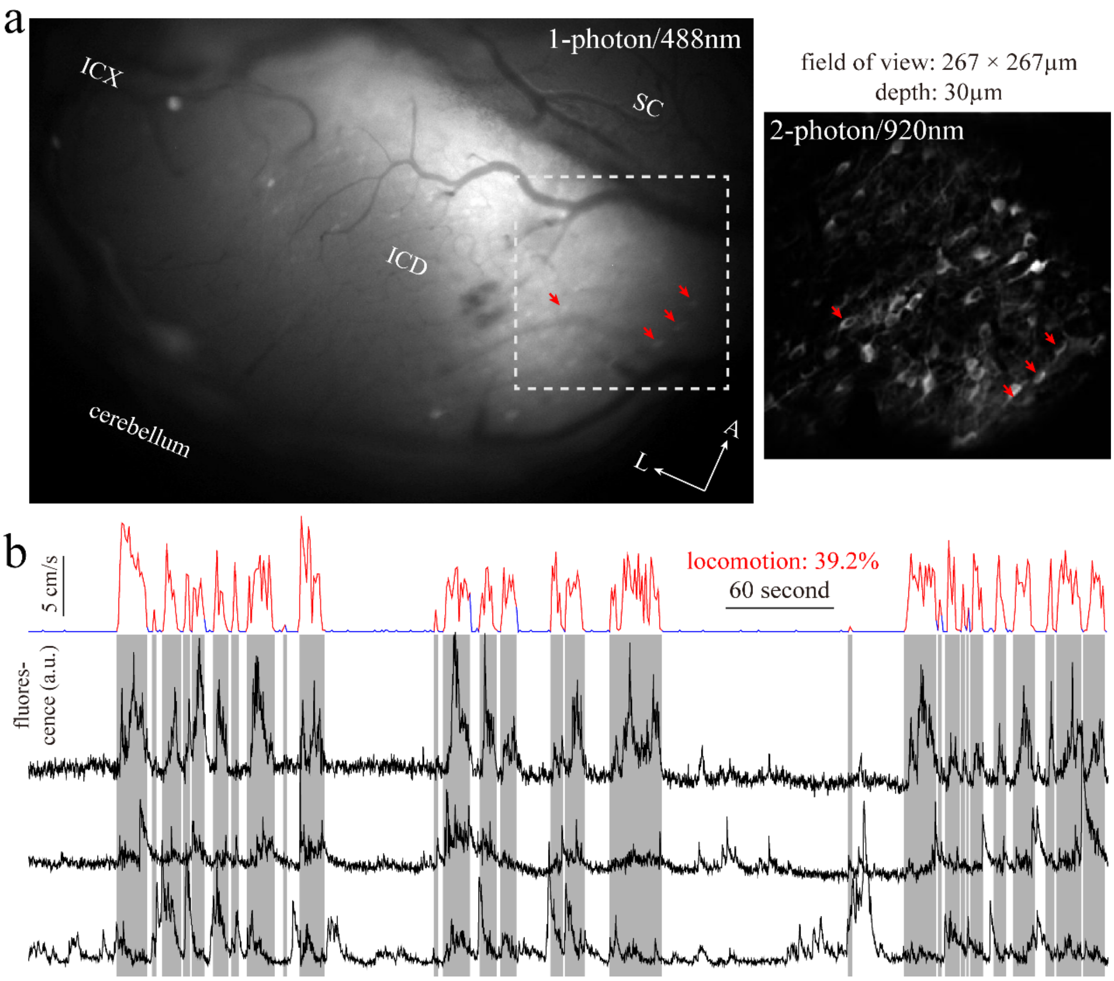

Supplementary Figure 2. Activities in the candidate ICD L1 of wild-type mice during locomotion. (a) FOV in the upright fluorescence microscope of awake mice candidate ICD L1 (left). FOV in the two-photon microscope (right). Noticed the four neurons that marked by red arrows in both figures. Same mouse as Supplementary Fig. 1a. Scale bar: 100 μm. (b) Locomotion traces (upper) and fluorescence of three neurons in the FOV (lower). The activities of first and third neurons were enhanced during locomotion. Raw data was uploaded (Supplementary Data 1).

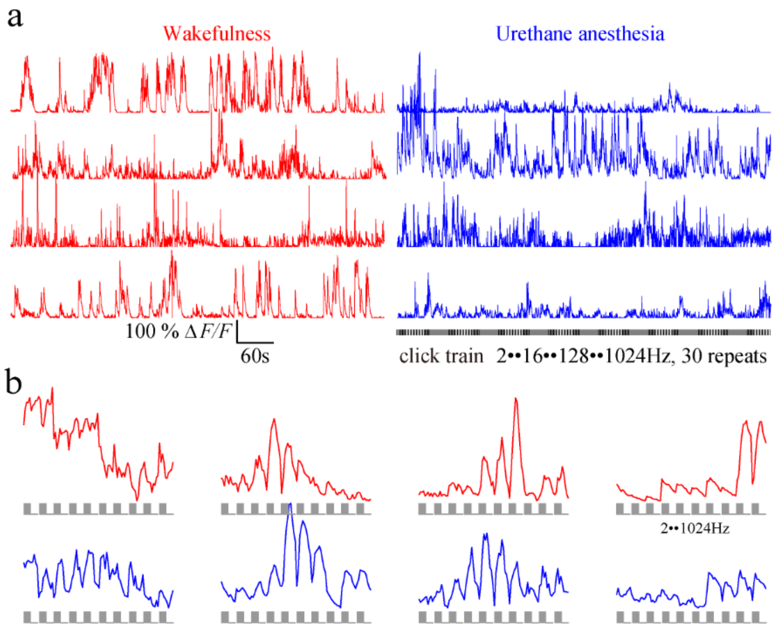

Supplementary Figure 3. The ΔF/F trace (i.e., relative fluorescence change) of neurons under wakefulness (red) and urethane anesthesia (blue) from different FOVs. (a) Raw ΔF/F traces of Fig 3c and d. The sound stimuli are randomized click trains. There are 10 different stimulus and each one is repeated 30 times. (b) Averaged ΔF/F traces of Fig 3c and d across 2 to 1024Hz temporal modulation frequency.

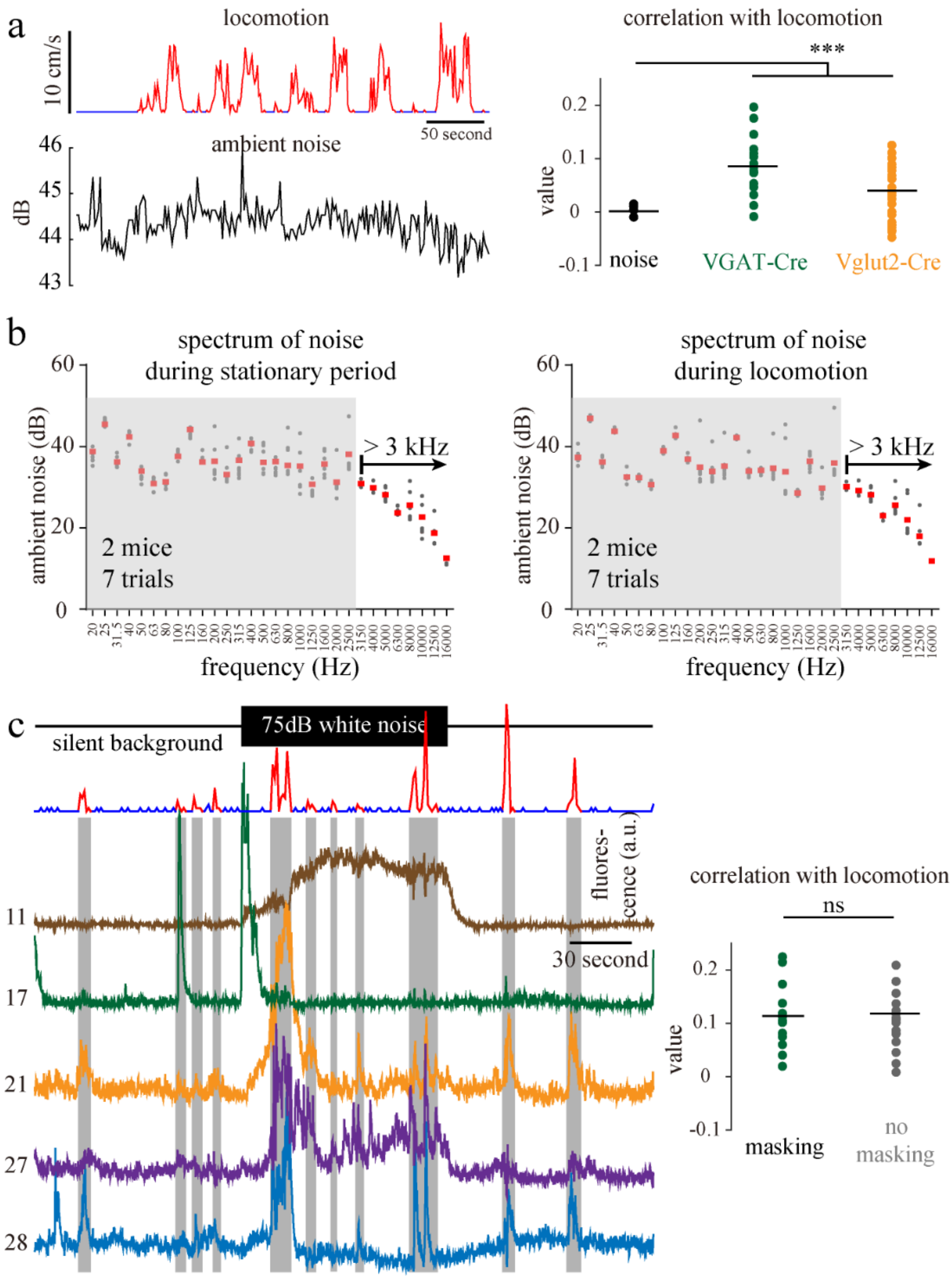

Supplementary Figure 4. Locomotion-related activities were unlikely to be an artifact of the running generated sound noise. (a) The correlation values between locomotion and noise, between locomotion and the neuronal activities of inhibitory and excitatory neurons. Red color was locomotion, blue color was stationary period (left-upper), ambient noise (left-lower) and correlation values (right). (b) The spectrum of background noise during stationary period and locomotion. The lowest spectral frequency used in our experiments is 3 kHz. Thus, we used arrow to highlight any frequency in the spectrum that was larger than 3 kHz, using gray area to highlight frequency that was less than 3 kHz. The single trial results were shown using gray dots, and the averaged results were shown using red dots. (c) Recording the spontaneously activities under wideband masking noise. The black lines and rectangular square showed silent and noise masking periods, respectively. The blue traces showed stationary periods, the red traces and grey bars showed locomotion. The different color of traces showed neuronal activities of five neurons (left). The correlation values between neuronal activities and locomotion under noise masking and without masking (right).

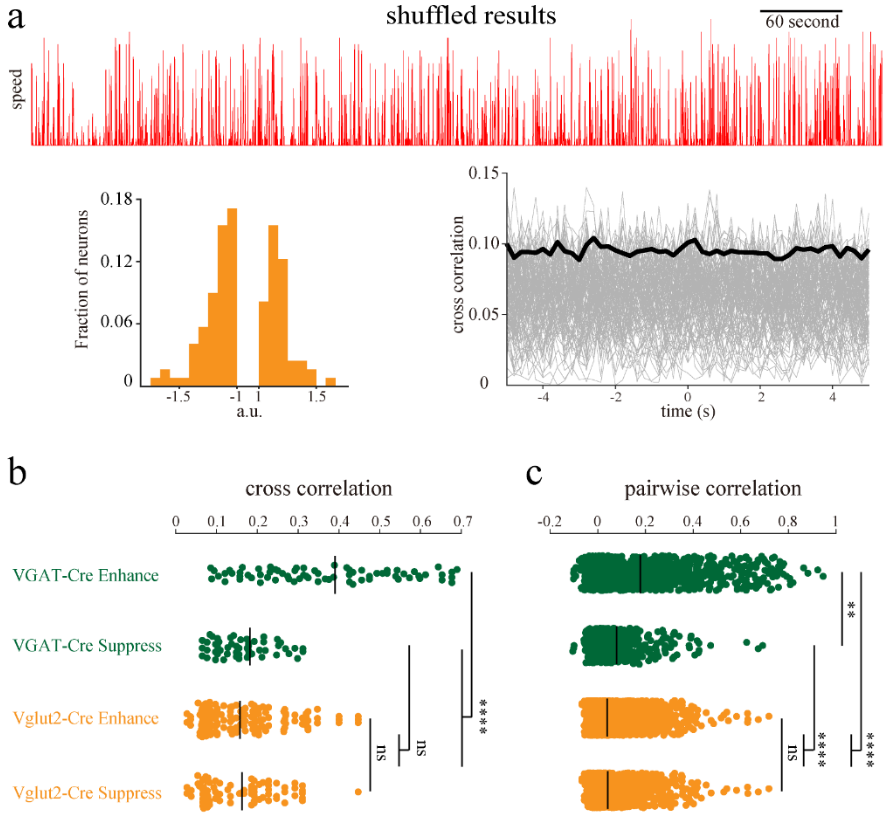

Supplementary Figure 5. Cross-correlation and pairwise correlation values of population neurons. (a) The shuffled locomotion traces of Fig. 5a (upper). Half of neurons showed enhanced or suppressed responses, but without anyone was modulated significantly (lower-left). Compared with Fig. 5e, the cross-correlation values after shuffling were distributed evenly (lower-right). (b) Cross-correlation between the locomotion-enhanced inhibitory neurons, locomotion-suppressed inhibitory neurons, locomotion-enhanced excitatory neurons, and locomotion-suppressed excitatory neurons with locomotion. (c) The pairwise correlations among the responses of 4 types of neurons. Kruskal-Wallis test with Dunn's multiple comparisons test. **p<0.01, ****p<0.0001, ns: not significant.

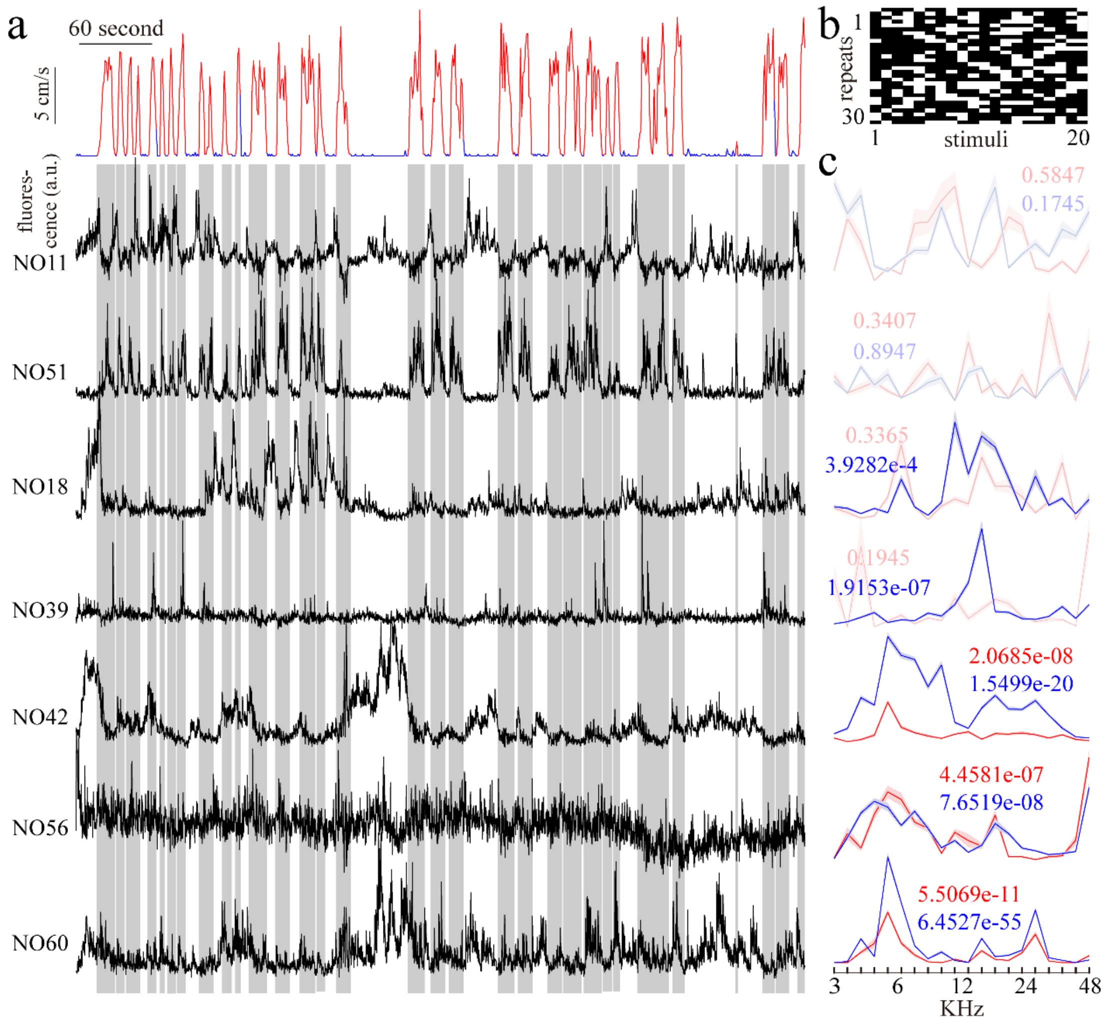

Supplementary Figure 6. Spectral tunings of excitatory neurons related to Fig. 6.

(a) Locomotion traces (upper). There are 81 locomotion events and 47.75% proportion of time is locomotion. Raw fluorescence of 7 simultaneously imaged excitatory neurons (lower). (b) The percentage of sound stimuli that belongs to the locomotion (white) and stationary periods (black). X-axis is 20 sound stimuli, and Y-axis is 30 repeats of each sound stimuli. The sound stimuli and locomotion occurred randomly. In this example, 202 sound stimuli coincided with the locomotion, i.e. 33.67% percentage of locomotion. (c) Spectral tunings under stationary periods (blue) and locomotion (red). The cases with no significant ($p>0.01$) tunings were displayed with dim colors. Raw data was uploaded (Supplementary Data 2).

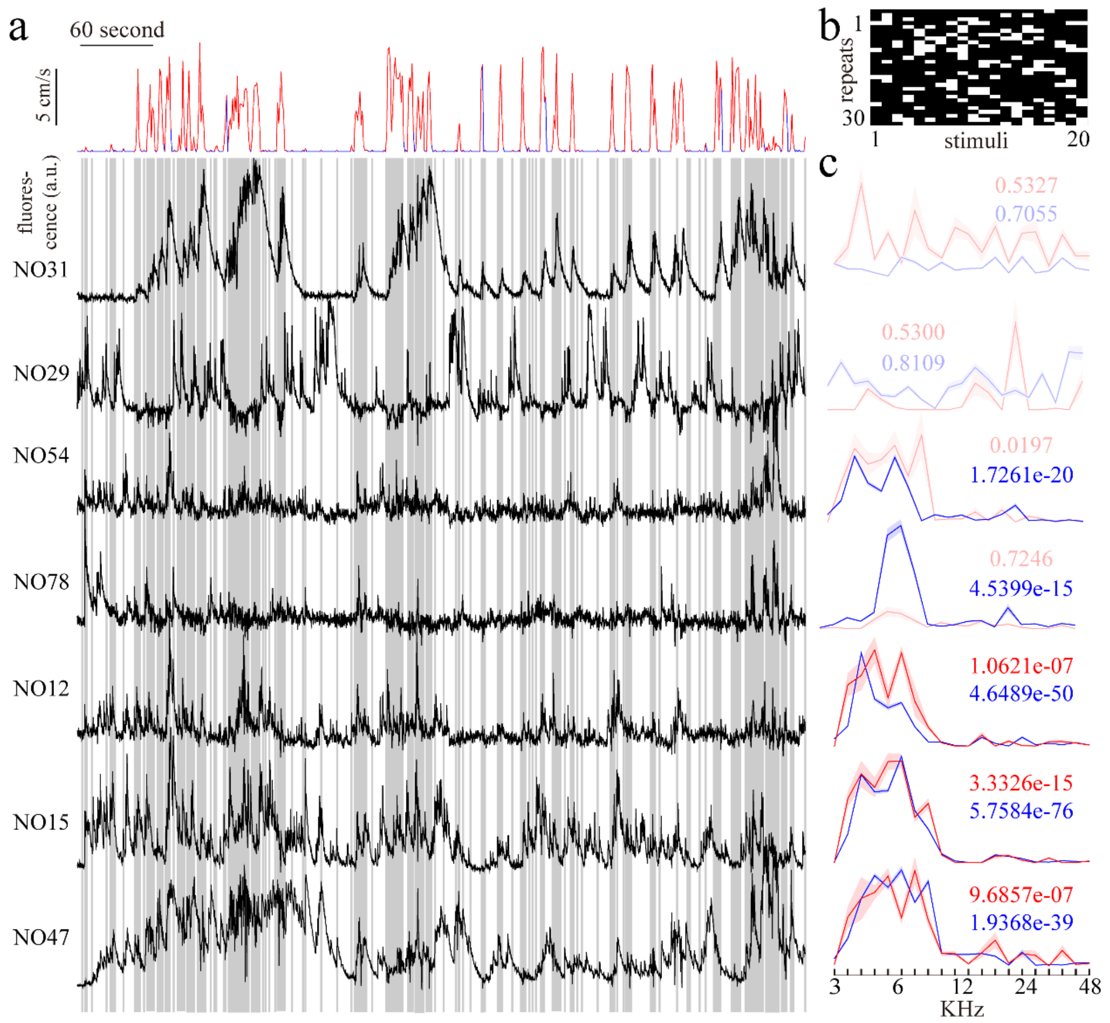

Supplementary Figure 7. Spectral tunings of inhibitory neurons related to Fig. 6.
(a) Locomotion traces (upper). There are 84 locomotion events and 36.94% proportion of time is locomotion. Raw fluorescence of 7 simultaneously imaged inhibitory neurons (lower). (b) 22.33% percentage of locomotion. (c) Spectral tunings of inhibitory neurons under stationary periods and locomotion. Raw data was uploaded (Supplementary Data 3).

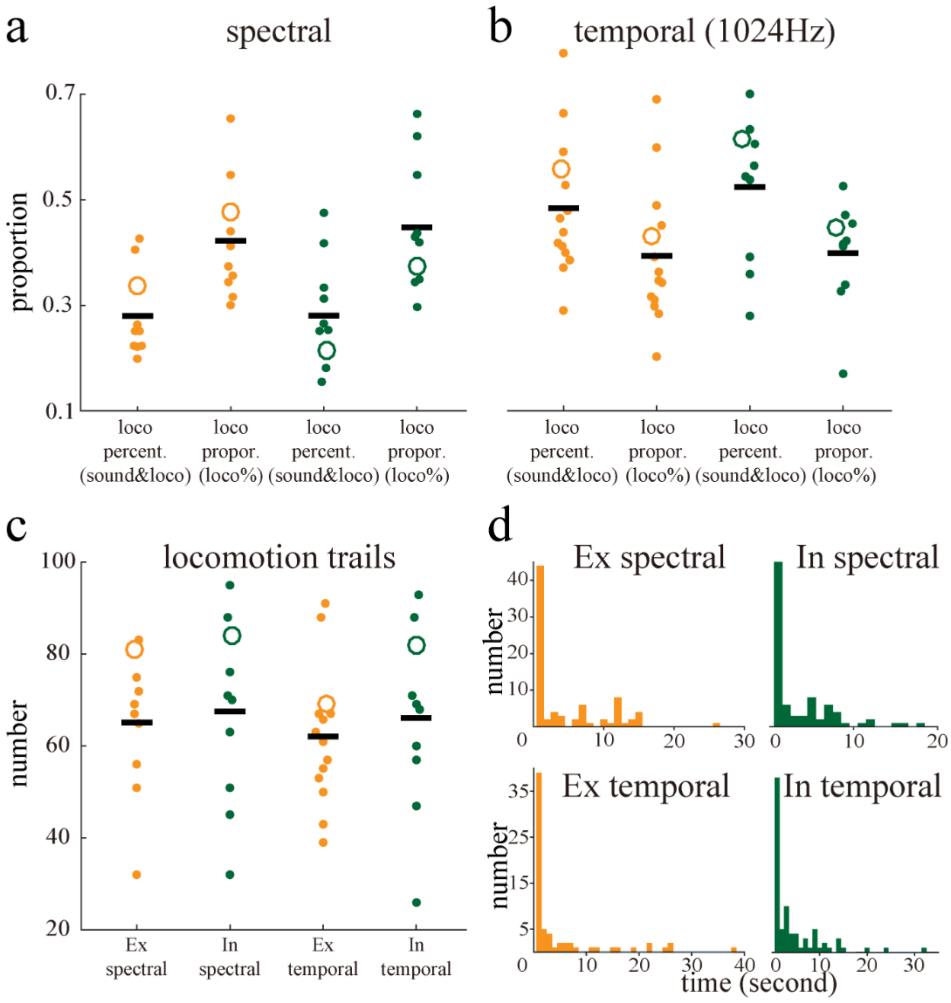

Supplementary Figure 8. Statistics of mice locomotion.

(a) Locomotion percentage (coincidence of sound stimuli and locomotion) and locomotion proportion (time during locomotion divide total time) for excitatory (yellow) and inhibitory (green) neurons under spectral tunings. The large dots represent Fig. Supplementary 6 and 7, respectively. (b) Similar as a but under the temporal tunings. The large dots represent Fig. Supplementary 9 and 10, respectively. Noticed that the locomotion percentage in a is lower than b, but the locomotion proportion is similar. It is because 50 ms spectral and 1000 ms temporal duration of sound are used in a and b, respectively. Therefore, the longer sound stimuli are more likely to coincide with the locomotion, which resulted in higher locomotion percentage. (c) The number of locomotion trails for excitatory and inhibitory neurons under spectral and temporal tunings. (d) The distribution of locomotion time for each trial showed in Supplementary 6, 7, 9 and 10.

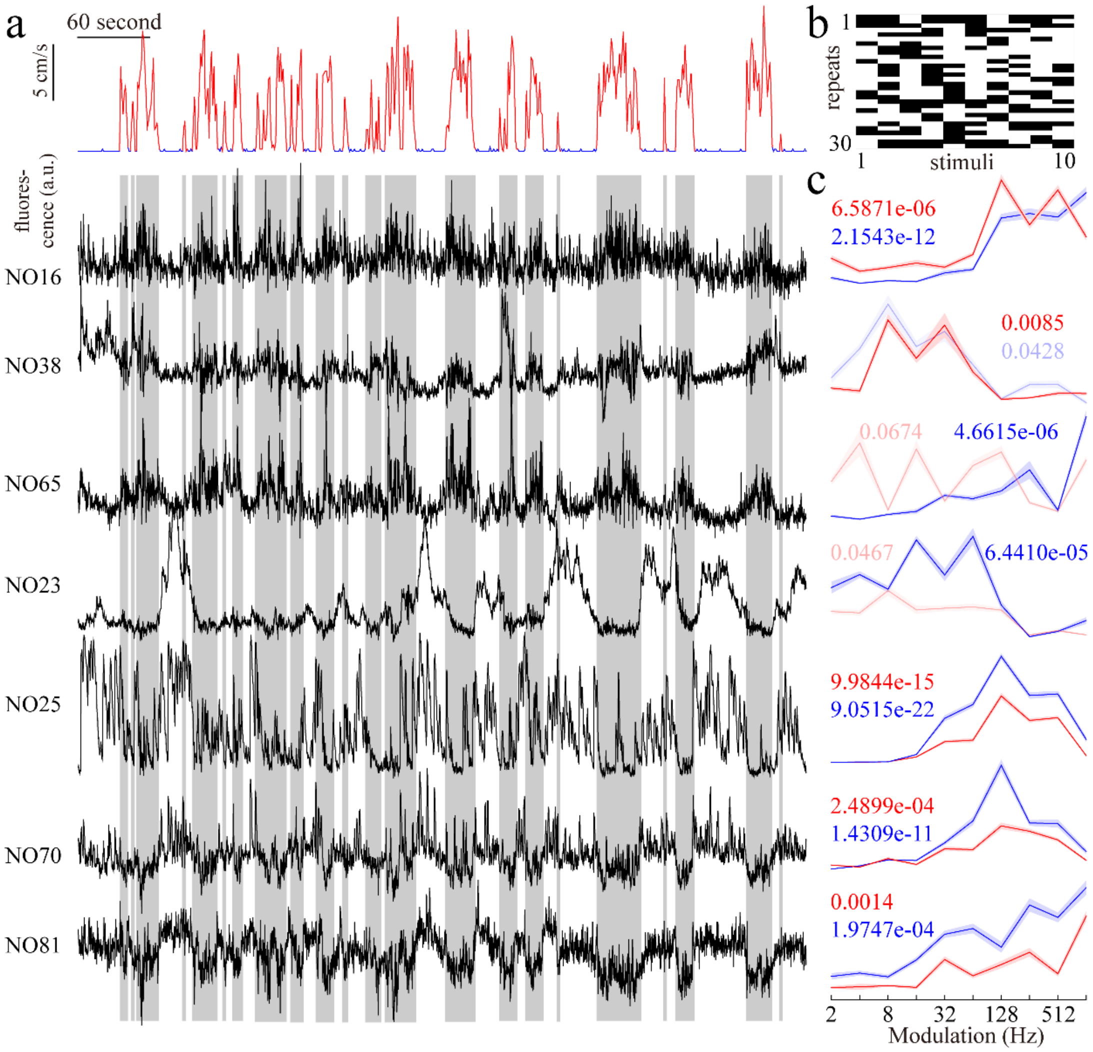

Supplementary Figure 9. Temporal tunings of excitatory neurons related to Fig. 7.
(a) Locomotion traces (upper). There are 67 locomotion events and 43.85% proportion of time is locomotion. Raw fluorescence of 7 simultaneously imaged excitatory neurons (lower). (b) 56.00 % percentage of locomotion. (c) Temporal tunings of excitatory neurons under stationary periods and locomotion. Raw data was uploaded (Supplementary Data 4).

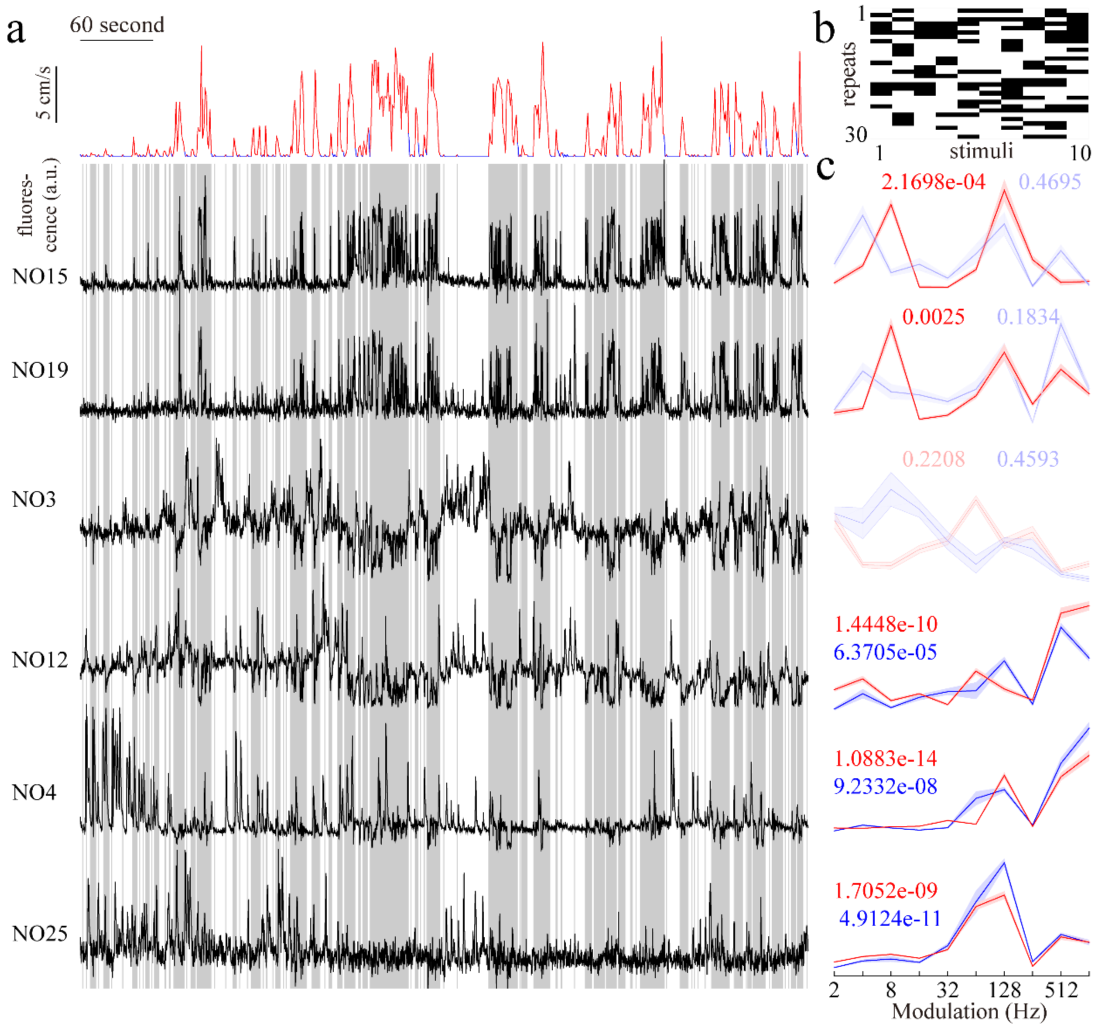

Supplementary Figure 10. Temporal tunings of inhibitory neurons related to Fig. 7.

(a) Locomotion traces (upper). There are 82 locomotion events and 45.35% proportion of time is locomotion. Raw fluorescence of 6 simultaneously imaged inhibitory neurons (lower). (b) 61.67 % percentage of locomotion. (c) Temporal tunings of inhibitory neurons under stationary periods and locomotion. NO4 and 25 show multi-peak tunings under two states, NO12 shows high-pass tuning during locomotion and multi-peak tuning during stationary periods. Raw data was uploaded (Supplementary Data 5).

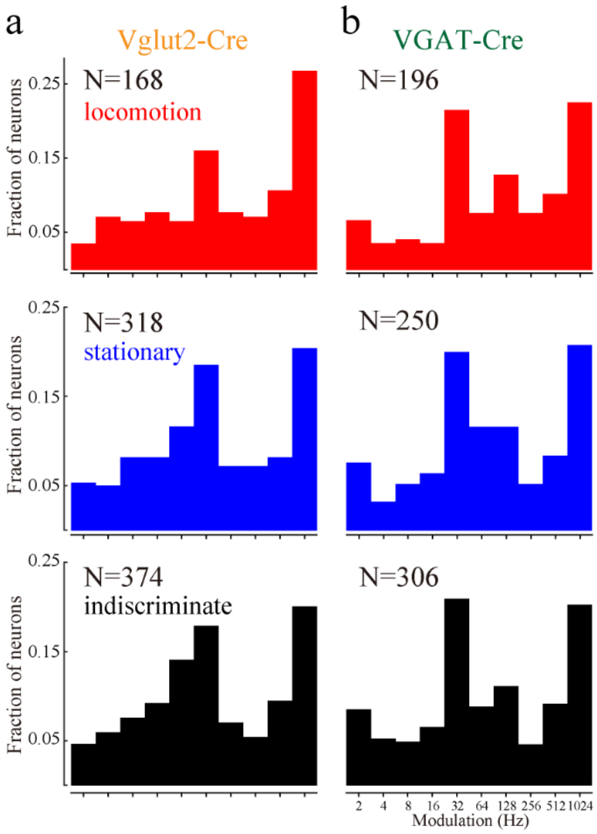

Supplementary Figure 11. The distribution of BMF.
Significantly tuned but unpaired neurons under locomotion and stationary periods (first and second rows), and locomotion indiscrimination neurons (third row). (a) Excitatory neurons. (b) Inhibitory neurons.

Supplementary Tables

	Low pass	High pass	Band reject	Band Pass	Multi Peak	Unchanged All types	Unchanged Band pass
2-1024Hz							
Vglut2-L/124	2(1.6)	26(21.0)	5(4.0)	37(29.8)	54(43.6)	70(56.5)	27(21.8)
Vglut2-S	3(2.4)	40(32.3)	2(1.6)	43(34.7)	36(29.0)		
VGAT-L/143	4(2.8)	34(23.8)	1(0.7)	52(36.4)	52(36.4)	99(69.2)	40(28.0)
VGAT-S	2(1.4)	38(26.6)	1(0.7)	57(40.0)	45(31.5)		
10-600Hz							
Vglut2-L/41	0(0)	1(2.4)	1(2.4)	14(34.2)	25(61.0)	31(75.6)	7(17.1)
Vglut2-S	0(0)	0(0)	0(0)	8(19.5)	33(80.5)		
VGAT-L/84	0(0)	0(0)	0(0)	24(28.6)	60(71.4)	63(75)	11(13.1)
VGAT-S	0(0)	1(1.2)	0(0)	19(22.6)	64(76.2)		

Supplementary Table 1. Response types of temporal tuning curves for neurons tuned under both stationary periods and locomotion. Each value is shown as number (percentage%).

Supplementary Note

For the long-range circuit, the ACx receives locomotion related inputs from motor cortex[1] and basal forebrain[2]. However, neither motor cortex nor basal forebrain project to the IC. Basal forebrain receives direct locomotion related inputs from the mesencephalic locomotor region (MLR), which includes the cuneiform nucleus (CnF) and pedunculopontine nucleus (PPN)[3]. Although our previous studies found that the excitatory neurons of IC shell receive higher proportion of projections from PPN than the inhibitory neurons [4], the PPN is unlikely contributes to the direct locomotion inputs of IC shell L1. First, if excitatory neuron was directly excited by PPN during locomotion, then its response will be highly correlated with the locomotion, instead of showing diverse and unreliable response (Fig. 5c). Second, glutamatergic neurons of CnF and PPN control locomotion[5], but Motts and Schofield observed that up to 89% of the PPN to IC projecting neurons are cholinergic positive[6]. Therefore, IC shell L1 may receive direct locomotion related inputs from CnF, SC, periaqueductal gray (PAG) or lateral paragigantocellular nucleus (LPGi) of brainstem, which are known for projecting to the IC and control locomotion[7]. In addition, the ACx feedback inputs may play an indirect role during locomotion.

For the local circuits, the disinhibitory model is a popular hypothesis in the VCx and ACx: during locomotion, excited VIP neurons will inhibit SOM neurons, thus disinhibiting principal excitatory neurons[2]. Since ACx was inhibited during locomotion, the disinhibitory effects on the ACx excitatory neurons might be counterbalanced by the dominate PV inhibitory inputs[1, 8]. In the IC shell, we propose that during locomotion, the locomotion directly excited inhibitory neurons (In-I) will inhibit one group of excitatory neurons (Ex-I), inhibit the other group of inhibitory neurons (In-II) and disinhibit the other group of excitatory neurons (Ex-II). Those four types of neurons may

represent the locomotion increased or decreased auditory responses of IC shell excitatory and inhibitory neurons (Fig. 8a). Lateral inhibition mechanisms may explain the tuning selectivity changes of IC shell excitatory and inhibitory neurons[9]. One study in the ACx revealed that lateral inhibition narrows the BW through the activation of SOM positive inhibitory neurons[10]. Thus, during locomotion, the direct and indirect long-range inputs will change the network activity of IC shell neurons. Those changes will enhance or suppress the lateral inhibition, thus narrow or broaden the BW for both excitatory and inhibitory neurons.

References

1. Schneider, D.M., Nelson, A. & Mooney, R. A synaptic and circuit basis for corollary discharge in the auditory cortex. *Nature* **513**, 189-194 (2014).
2. Fu, Y. *et al.* A Cortical Circuit for Gain Control by Behavioral State. *Cell* **156**, 1139-1152 (2014).
3. Lee, A.M. *et al.* Identification of a Brainstem Circuit Regulating Visual Cortical State in Parallel with Locomotion. *Neuron* **83**, 455-466 (2014).
4. Chen, C.G., Cheng, M.X., Ito, T. & Song, S. Neuronal Organization in the Inferior Colliculus Revisited with Cell-Type-Dependent Monosynaptic Tracing. *J Neurosci* **38**, 3318-3332 (2018).
5. Caggiano, V. *et al.* Midbrain circuits that set locomotor speed and gait selection. *Nature* **553**, 455-460 (2018).
6. Motts, S.D. & Schofield, B.R. Sources of Cholinergic Input To the Inferior Colliculus. *Neuroscience* **160**, 103-114 (2009).
7. Gatto, G. & Goulding, M. Locomotion Control: Brainstem Circuits Satisfy the Need for Speed. *Curr Biol* **28**, R256-R259 (2018).
8. Zhou, M. *et al.* Scaling down of balanced excitation and inhibition by active behavioral states in auditory cortex. *Nat Neurosci* **17**, 841-850 (2014).
9. Isaacson, J.S. & Scanziani, M. How Inhibition Shapes Cortical Activity. *Neuron* **72**, 231-243 (2011).
10. Kato, H.K., Asinof, S.K. & Isaacson, J.S. Network-Level Control of Frequency Tuning in Auditory Cortex. *Neuron* **95**, 412-423 (2017).
