# Supplementary material for: Differential cell-type dependent brain state modulations of sensory representations in the non-lemniscal mouse inferior colliculus
Source: Commun Biol. 2019 Sep 30;2:356. doi: 10.1038/s42003-019-0602-4 (PMC6769006; doi:10.1038/s42003-019-0602-4)
Supplement: Supplementary file 2 — Description of Additional Supplementary Files [file 42003_2019_602_MOESM2_ESM.docx]

Description of Additional Supplementary Files

**File Name**: Supplementary Data 1

**Description**: Supplementary Fig. 2 Raw Data

**File Name**: Supplementary Data 2

**Description**: Supplementary Fig. 6 Raw Data

**File Name**: Supplementary Data 3

**Description**: Supplementary Fig. 7 Raw Data

**File Name**: Supplementary Data 4

**Description**: Supplementary Fig. 9 Raw Data

**File Name**: Supplementary Data 5

**Description**: Supplementary Fig. 10 Raw Data
